# Developing a Personalized Cancer Nanovaccine Using Coxsackievirus‐Reprogrammed Cancer Cell Membranes for Enhanced Anti‐Tumor Immunity

**DOI:** 10.1002/advs.202506791

**Published:** 2025-07-29

**Authors:** Amirhossein Bahreyni, Yasir Mohamud, Amrit Singh, Razieh Sadat Banijamali, Jeffrey Tang, Jingchun Zhang, Honglin Luo

**Affiliations:** ^1^ Centre for Heart Lung Innovation St Paul's Hospital Vancouver BC V6Z 1Y6 Canada; ^2^ Department of Pathology and Laboratory of Medicine University of British Columbia Vancouver BC V6Z 1Y6 Canada; ^3^ Department of Anesthesiology, Pharmacology and Therapeutics University of British Columbia Vancouver BC V6Z 1Y6 Canada

**Keywords:** breast cancer, cancer immunotherapy, coxsackievirus B3 (CVB3), nanoparticle, personalized cancer vaccine, reprogramming cancer cell membranes

## Abstract

Cancer vaccines emerge as a promising approach in immunotherapy, but their efficacy is often hindered by immunosuppressive factors like PD‐L1 on tumor cell membranes. To address this challenge, a personalized nanovaccine is developed using membranes from Coxsackievirus B3 (CVB3)‐infected 4T1 breast cancer cells combined with heat‐deactivated CVB3 (hdCVB3) encapsulated in PLGA nanoparticles (PLGA@hdCVB3I4T1M). RNA sequencing reveals significant upregulation of immune activation‐related genes, while protein analysis demonstrates reduced immunosuppressive markers (PD‐L1, B7‐H3, CD47) and increased immunostimulatory proteins (calreticulin), enhancing immune cell uptake and activation. In vitro and in vivo studies confirm the safety and potent immunostimulatory effects of PLGA@hdCVB3I4T1M, leading to enhanced immune cell infiltration, elevated proinflammatory cytokine production, and robust antitumor responses. The nanovaccine significantly improves tumor suppression and prolongs survival in animal models. Additionally, the inclusion of hdCVB3 amplified immune recognition of both viral and tumor antigens, further enhancing therapeutic efficacy, particularly when combined with oncolytic virotherapy. Mechanistically, this strategy primes the immune system for a more effective and sustained antitumor response. In summary, PLGA@hdCVB3I4T1M effectively stimulates the immune system, overcoming tumor immune evasion. This nanovaccine represents a promising strategy for enhancing cancer immunotherapy and holds strong potential for clinical translation, particularly in combination with oncolytic virotherapy.

## Graphical abstract

A personalized nanovaccine combining PLGA‐encapsulated heat‐inactivated CVB3 and membranes from CVB3‐infected breast cancer cells stimulates potent antitumor immunity. It reduces immunosuppressive markers, enhances immune activation, and improves survival in vivo. This platform offers a promising strategy to overcome immune evasion and can be combined with oncolytic virotherapy for enhanced therapeutic outcomes.

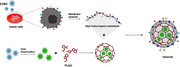

## 1 Introduction

Breast cancer is the most common cancer in women, with high incidence and significant mortality rate.^[^ ^1^ ^]^ Triple‐negative breast cancer (TNBC), which lacks estrogen receptor, progesterone receptor, and human epidermal growth factor‐2 (HER‐2) receptors, is the most aggressive subtype, accounting for 20% of cases and having the worst prognosis due to its rapid progression and high metastatic potential.^[^ ^2^ ^]^ Standard treatments, including surgery, radiotherapy, and chemotherapy, often yield poor outcomes for TNBC patients, highlighting the need for new therapeutic options.^[^ ^3^ ^]^

Cancer immunotherapy harnesses the immune system to detect and eliminate cancer cells, and among its various approaches, cancer vaccines have attracted significant attention.^[^ ^4^ ^]^ These vaccines aim to stimulate an immune response against tumor cells by introducing cancer‐specific antigens.^[^ ^5^, ^6^ ^]^ However, developing an effective cancer vaccine remains challenging, as it requires an optimal source of tumor antigens, immune‐stimulating adjuvants, and a well‐designed delivery platform.^[^ ^7^ ^]^ Earlier studies have shown that using a single tumor antigen often fails to robustly activate the immune system against cancer cells.^[^ ^8^, ^9^, ^10^ ^]^ To overcome this limitation, whole cell lysate‐based vaccines have been explored, offering a broader range of antigens. However, these vaccines typically provide limited therapeutic benefits due to interference from non‐relevant cellular components, such as housekeeping proteins, carbohydrates, and lipids.^[^ ^11^ ^]^ Another approach involves membrane‐based cancer vaccines, which use tumor cell membranes as a source of antigens. While this strategy avoids some of the pitfalls associated with whole cell lysates, the overexpression of immune checkpoints, such as programmed death‐ligand 1 (PD‐L1), on the membranes of cancer cells and the lack of ligands for targeting antigen‐presenting cells (APCs) can inhibit effective immune activation, limiting the potential of membrane‐based vaccines to induce a robust anti‐tumor response.^[^ ^12^, ^13^, ^14^ ^]^ Therefore, to elicit robust immune responses, it is crucial to enhance the immunogenicity of cancer cell membranes, ensuring their effectiveness as a potent cancer vaccine.

Coxsackievirus B3 (CVB3) is a non‐enveloped, single‐stranded RNA virus belonging to the enterovirus genus within the Picornaviridae family. While it is primarily known for causing viral myocarditis and pancreatitis, CVB3 has also been studied for its oncolytic potential, particularly in targeting cancer cells.^[^ ^15^, ^16^ ^]^ During the infection phase, prior to cell lysis, CVB3 alters the expression of various cellular proteins, including those involved in immune regulation.^[^ ^17^, ^18^ ^]^ This viral modulation impacts immune‐related pathways, potentially enhancing the immunogenicity of the infected cancer cells. Studies have demonstrated that the administration of wild‐type CVB3 (WT‐CVB3) for tumor treatment in vivo can function as a natural vaccine, primarily by inducing immunogenic cell death and significantly enhancing immune cell infiltration into the tumor microenvironment.^[^ ^15^, ^19^ ^]^ However, the use of WT‐CVB3 to generate in situ vaccines is limited due to the substantial toxicity it can cause, particularly cardiotoxicity and pancreatoxicity.^[^ ^20^ ^]^ To address this challenge, we developed an in vitro vaccine by infecting cancer cells with CVB3 and isolating the membranes from these infected cells. This strategy enables the incorporation of tumor antigens onto the membranes while simultaneously allowing CVB3 to exert effects on immunoregulatory proteins on the membrane surface, potentially creating an effective immunogen for activating immune cells.

Nanoparticles are widely utilized for the delivery of drugs to various organs and play a crucial role in cancer vaccine development.^[^ ^21^ ^]^ Their application is essential for preventing the degradation of neoantigens, ensuring their stability and bioavailability. Additionally, nanoparticles provide the opportunity to combine tumor antigens with adjuvants, thereby enhancing immune activation and improving the overall efficacy of cancer vaccines.^[^ ^22^, ^23^ ^]^ PLGA (Poly(lactic‐co‐glycolic acid)) is a widely utilized biocompatible and biodegradable copolymer that plays a significant role in the formulation of cancer vaccines.^[^ ^24^ ^]^ Its inherent ability to encapsulate tumor antigens facilitates controlled release and protects these antigens from degradation, thereby enhancing immune activation and optimizing the therapeutic efficacy of cancer immunotherapies.^[^ ^25^ ^]^ Furthermore, extensive studies have demonstrated that PLGA exhibits immune adjuvant properties, further establishing its suitability as a valuable candidate for advanced vaccine development.^[^ ^26^ ^]^

In this study, we demonstrate that infecting cancer cells with CVB3 significantly alters the immunological properties of their membranes, rendering them more immunostimulatory for immune cell activation against cancer cells. Specifically, our RNA sequencing analysis reveals that CVB3 infection upregulates immunostimulatory genes, further supporting its role in reshaping the tumor microenvironment. Additionally, CVB3 infection markedly reduces the expression of immune checkpoint proteins, such as B7 homolog 3 (B7‐H3), PD‐L1, and CD47, while increasing the levels of immunostimulatory proteins like calreticulin on the membrane of cancer cells. The membranes isolated from these infected cancer cells not only retain tumor antigens but also exhibit a favorable profile characterized by lower levels of immune‐suppressive proteins and higher levels of immune‐stimulatory proteins, thereby promoting more vigorous activation of the immune system.

Building upon these observations, we developed a personalized nanovaccine by coating PLGA nanoparticles with membranes derived from CVB3‐infected tumor cells. Previous studies have shown that vaccines containing adjuvants possess enhanced potential to stimulate APCs, leading to the expression of co‐stimulatory signals and the release of cytokines, thereby broadening the immune response.^[^ ^27^ ^]^ To further maximize immune cell activation, we loaded PLGA nanoparticles, coated with membranes from infected cells (PLGA@I4T1M), with heat‐deactivated CVB3 (hdCVB3), resulting in a formulation called PLGA@hdCVB3I4T1M. This approach not only amplifies the immune response against cancer cells but also significantly increases immune cell infiltration into the tumor microenvironment when combined with a safe, attenuated form of CVB3—microRNA‐modified CVB3 (miR‐CVB3) ^[^ ^15^ ^]^—used as an oncolytic virus following vaccination. Hence, herein we first evaluated the efficacy of the personalized nanovaccine (PLGA@hdCVB3I4T1M) in activating the immune system and inhibiting tumor growth through a series of in vitro and in vivo experiments. Subsequently, we assessed the impact of combining PLGA@hdCVB3I4T1M with miR‐CVB3 as a viro‐immunotherapy approach in tumor‐bearing mice.

## 2 Experimental Section

### 2.1 Cell Culture

The 4T1 cells (CRL‐2539, a murine triple‐negative mammary tumor cell line derived from Balb/c mice), RAW 264.7 cells (TIB‐71, a macrophage‐like cell line from Balb/c mice), HeLa cells (CCL‐2, human cervical cancer cell line), and MCF‐10‐A cells (CRL‐10317, a human normal mammary epithelial cell line) were obtained from the American Type Culture Collection (ATCC). Both 4T1 and RAW 264.7 cells were cultured in Roswell Park Memorial Institute (RPMI) 1640 medium supplemented with 10% fetal bovine serum (FBS) and 1% antibiotics (100 µg mL^−1^ streptomycin and 100 U mL^−1^ penicillin). HeLa cells were maintained in Dulbecco's Modified Eagle's Medium (DMEM) containing 10% FBS and 1% antibiotics (100 µg mL^−1^ streptomycin, 100 U mL^−1^ penicillin). MCF‐10‐A cells were cultured in DMEM/F12 medium supplemented with 10% FBS and 1% antibiotics (100 µg mL^−1^ streptomycin, 100 U mL^−1^ penicillin).

### 2.2 Animal

Female Balb/c mice (000651, The Jackson Laboratory), aged 6 to 8 weeks, were used for the in vivo experiments. All animal procedures were conducted in strict accordance with the ethical guidelines for the care and use of laboratory animals and were approved by the Animal Care Committee at the University of British Columbia (A22‐0237).

### 2.3 Generation of Recombinant CVB3 and Virus Propagation

The miR‐CVB3 was engineered as previously described.^[^ ^28^ ^]^ Briefly, four copies of the miRNA‐145 target sequence (TS), four copies of the miRNA‐216 TS, two copies of the miRNA‐1 TS, and two copies of the miRNA‐143 TS were inserted into the 5′ untranslated region of the CVB3 genome. Both recombinant CVB3 (miR‐CVB3) and WT‐CVB3 were propagated in HeLa cells and stored at −80 °C for future use.

### 2.4 RNA Extraction and RNA Sequencing

4T1 cells were infected with CVB3 at a multiplicity of infection (MOI) of 1. Following 6 h incubation, total RNA was extracted from both CVB3‐infected and non‐infected 4T1 cells using the RNeasy Mini Kit (74104, Qiagen) following the manufacturer's protocol. RNA quantity and quality were assessed using a NanoDrop spectrophotometer (Thermo Fisher Scientific). Library preparation and RNA sequencing (RNA‐seq) were performed by Illumina. Briefly, RNA samples were processed for mRNA enrichment, and sequencing libraries were prepared using the TruSeq Stranded mRNA Library Prep Kit (Illumina). The sequencing was performed on an Illumina NovaSeq 6000 platform, generating 150 bp paired‐end reads. Sequencing data were processed using FastQC (v0.11.8) for quality control.

### 2.5 RNA‐Seq Data Analysis

Transcript quantification was performed using Salmon (v1.7.0) using the Mouse Release M36 (GRCm39) transcriptome. Alignment to the CVB3 virus genome (strain Nancy) was performed using HISAT2 (v2.1.0). Transcripts with a minimum of 5 counts across all samples were retained for downstream analysis. Differential expression analysis was performed using limma voom (v3.60.6). A Benjamini–Hochberg false discovery rate (FDR) of 5% was used to identify differentially expressed gene‐transcripts. Gene set analysis was performed using enrichR (v3.2) using WikiPathways (2024 Mouse) and KEGG (2029 Mouse) databases. All analyses were performed using the statistical computing program (v4.1.4) and RStudio (v2023.09.1+494).

### 2.6 Virus Infection and Isolation of Cancer Cells Membranes

To generate membranes from infected cancer cells, 4T1 cells were infected with CVB3 at an MOI of 1. Following an overnight infection, the cells were collected and dispersed in deionized water. The cells were then homogenized on ice to ensure complete disruption of the cells. The mixture was initially centrifuged at 4 °C (10^4^ × g, 30 min) to remove organelles and larger cell fragments. The membranes were then separated from the supernatant by a subsequent high‐speed centrifugation (10^5^× g, 2 h). The isolated membranes (I4T1M) were resuspended in phosphate‐buffered saline (PBS) and stored at −80 °C. Control membranes from uninfected 4T1 cells (4T1M) were isolated using the same procedure.

### 2.7 Assessment of the Impact of CVB3 on Membrane Proteins and Immunogenicity of Infected Membrane

To evaluate the impact of CVB3 infection on immunological proteins expressed on the membrane of cancer cells, a panel of key proteins including PD‐L1, B7‐H3, CD47, CD39, and calreticulin was analyzed using western blotting. Specifically, ≈20 µg of protein from both infected and non‐infected cell membranes were separated using 12% SDS‐PAGE (sodium dodecyl sulfate‐polyacrylamide gel electrophoresis). The proteins were then transferred to a polyvinylidene fluoride membrane, blocked for 1 h with a 5% skimmed milk solution, and incubated overnight at 4 °C with the following primary antibodies: anti‐PD‐L1 (A1645, ABclonal), anti‐B7‐H3 (A17216, ABclonal), anti‐calreticulin (A11563, ABclonal), anti‐CD47 (A21904, ABclonal), anti‐VP1 (M47, Mediagnost), and anti‐CD39 (A3778, ABclonal). Afterward, the membranes were washed three times with Tris‐buffered saline with Tween (TBST) and incubated with secondary antibodies for 1 h at room temperature. Finally, chemiluminescent imaging and NIH ImageJ (version 1.52p) analysis were conducted to visualize and quantify protein expression, respectively.

To further assess the immune‐stimulatory potential of the infected and non‐infected membranes, RAW 264.7 macrophages were treated with membrane preparations, and the expression of key inflammatory genes (*tnf‐α*, *cxcl10*, and *ccl5*) was measured using reverse transcription quantitative PCR (RT‐qPCR). 2 h post‐incubation, total RNA was extracted using the RNeasy Mini Kit (74104, Qiagen), and 1 µg of RNA was used for the qPCR reaction with the TaqMan RNA‐to‐CT 1‐Step Kit (4392653, Thermo Fisher Scientific) on a ViiA 7 Real‐Time PCR System (Applied Biosystems). Gene expression data were normalized to *β‐actin* mRNA levels, and the comparative CT (2‐ΔΔCT) method was applied to calculate relative fold changes. All reactions were performed in triplicate, and the primer sequences used are detailed in **Table** **1**.

**Table 1:** Primers used for the RT‐qPCR analysis.

Target	Forward	Reverse
Murine *tnf‐α*	5’‐ GTC CCC AAA GGG ATG AGA AGT T ‐3’	5’‐ GTT TGC TAC GAC GTG GGC TAC A ‐3’
Murine *cxcl10*	5’‐ GCT GGG ATT CAC CTC AAG AA ‐3’	5’‐ CTT GGG GAC ACC TTT TAG CA ‐3’
Murine *ccl5*	5’‐ GCT TTG CCT ACC TCT CC ‐3’	5’‐ TCG AGT GAC AAA CAC GAC TGC ‐3’
Murine *ifnb1*	5′‐CTT GGA TTC CTA CAA AGA AGC AGC‐3′	5′‐TCC TCC TTC TGG AAC TGC TGCA‐3′
Murine *β‐actin*	5′‐CAT TGC TGA CAG GAT GCA GAA GG‐3′	5′‐TGC TGG AAG GTG GAC AGT GAG G‐3′

Additionally, the activation of RAW 264.7 macrophages was assessed after 12 h of incubation with membrane preparations. The cells were washed three times with PBS and stained with CD80‐PE (B340153, BioLegend) and MHCII–Alexa Fluor 647 (B346505, BioLegend) for 30 min. The activation status was then analyzed by flow cytometry, and the data were processed using FlowJo version 10. This approach allowed for a comprehensive assessment of how CVB3 infection influences immune‐related proteins on the cancer cell membrane and their subsequent effect on macrophage activation.

To evaluate the effect of CVB3‐infected tumor membranes on dendritic cell activation, bone marrow‐derived dendritic cells (BMDCs) were isolated from the femurs and tibias of naïve Balb/c mice (6–8 weeks old). Briefly, bone marrow cells were flushed from the bones with RPMI‐1640 medium supplemented with 10% FBS, filtered through a 70 µm cell strainer, and cultured in six‐well plates at a density of 1 × 10⁶ cells mL^−1^ in RPMI‐1640 containing 10% FBS, 20 ng mL^−1^ GM‐CSF (Stemcell Technologies), and 10 ng mL^−1^ IL‐4 (Stemcell Technologies). Dendritic cells were treated with membrane preparations for 12 h. Following incubation, cells were washed three times with PBS and stained for 30 min at 4 °C with anti‐CD80‐PE (B340153, BioLegend) and anti‐MHC class II–Alexa Fluor 647 (B346505, BioLegend) to assess activation status. Samples were acquired on a flow cytometer, and data were analyzed using FlowJo version 10.

To assess tumor‐specific T cell responses elicited by the I4T1M and 4T1M vaccines, tumor‐free Balb/c mice (*n* = 3 per group) were immunized subcutaneously with 100 µg of either I4T1M or 4T1M on days 0 and 7. One week after the final vaccination, spleens were harvested, and single‐cell suspensions were prepared following red blood cell lysis. Isolated splenocytes (1 × 10⁶ cells/well) were restimulated ex vivo with lysates from 4T1 tumor cells for 72 h. Supernatants were then collected, and levels of IFN‐γ and TNF‐α were quantified using ELISA kits (ab100689 and ab208348, Abcam), following the manufacturer's instructions.

### 2.8 Preparation and Characterization of the Personalized Cancer Vaccine

A personalized cancer vaccine was formulated using a modified water‐oil‐water (w/o/w) double emulsion and solvent evaporation technique.^[^ ^29^ ^]^ In this process, hdCVB3 was first dissolved in sodium acetate buffer. This solution was gradually added to a dichloromethane mixture containing 20 mg of PLGA (Sigma–Aldrich), and the suspension was subjected to probe sonication for 10 min on ice to form the primary emulsion. The resulting mixture was then emulsified into a 2% polyvinyl alcohol (PVA) solution (Sigma–Aldrich) and probe sonicated again for 15 min on ice to create the secondary emulsion. The PLGA@hdCVB3 nanoparticles were collected by centrifugation at 15 000 rpm for 30 min, washed three times with deionized water, and resuspended. To coat the nanoparticles with I4T1M, an equal amount of membrane and PLGA@hdCVB3 nanoparticles were mixed together and magnetically stirred overnight at 4 °C, yielding the final nanocomplex, PLGA@hdCVB3I4T1M. For the characterization, the size and zeta potential of the PLGA@hdCVB3 and PLGA@hdCVB3I4T1M were measured by dynamic light scattering (DLS, Malvern Zetasizer). The morphology of the final nanoparticles (PLGA@hdCVB3I4T1M) was evaluated using a transmission electron microscopy (TEM).

### 2.9 Cytotoxicity Study

For the cytotoxicity assay, 4T1 and MCF‐10A cells were seeded into 96‐well plates at a density of 1 × 10⁴ cells per well. Cells were treated with different formulations at varying concentrations and incubated for 36 h. After incubation, MTS reagent (3‐(4,5‐dimethylthiazol‐2‐yl)‐5‐(3‐carboxymethoxyphenyl)‐2‐(4‐sulfophenyl)‐2H‐tetrazolium, G3582, Promega) was added to each well, followed by an additional 3 h incubation. Cell viability was then assessed by measuring the absorbance at 490 nm using a BioTek Synergy H1 microplate reader. Untreated cells were used as a control, representing 100% cell viability. The percentage of cell inhibition was calculated relative to this baseline.

### 2.10 In vitro Macrophage Maturation Assays and Repolarization

To evaluate the effectiveness of the developed personalized cancer vaccine in activating APCs, RAW 264.7 macrophages (5 × 10^5^ cells) were incubated for 12 h with various vaccine formulations at a final concentration of 100 µg mL^−1^. Following this incubation, the cells were washed three times with PBS and stained for 30 min with CD80‐PE (B340153, BioLegend) and MHCII–Alexa Fluor 647 (B346505, BioLegend). The activation status of the macrophages was subsequently assessed using flow cytometric analysis, with the acquired data analyzed using FlowJo version 10 software. The activation of BMDCs treated with the same formulations was also evaluated by flow cytometric analysis using the same markers. The expression level of COX‐2 in macrophages was assessed after a 12 h incubation with various formulations using a COX‐2 antibody (12282, Cell Signaling Technology) and western blot analysis. Additionally, the gene expression levels of *tnf‐α*, *ifnb1*, and *ccl5* were evaluated via RT‐qPCR after incubating the macrophages with the different vaccine formulations as described above.

To assess the repolarization effect of the vaccine on alternatively activated (M2‐like) macrophages, RAW 264.7 cells were seeded into 6‐well plates at a density of 1 × 10⁶ cells per well. Cells were then polarized to an M2 phenotype by treatment with interleukin‐4 (IL‐4, 100 ng mL^−1^) for 24 h. After polarization, cells were washed three times with PBS to remove residual IL‐4 and replenished with 2 mL of fresh culture medium containing either PBS (control) or the PLGA@hdCVB3I4T1M vaccine. Following 24 h of treatment, cells were collected, washed with PBS, and stained simultaneously with anti‐CD80‐PE (B340153, BioLegend) and anti‐CD206‐Alexa Fluor 647 (141712, BioLegend) antibodies for 30 min on ice in the dark. After staining, cells were washed and analyzed using a flow cytometer. Data were processed and quantified using FlowJo version 10 software.

### 2.11 In Vivo Safety Assessment of Vaccines

Six to eight‐week‐old Balb/c mice were divided into four groups (*n* = 5 per group). The first group received PBS as a control, while the remaining groups received three subcutaneous injections of different vaccine formulations (PLGA@hdCVB3, PLGA@I4T1M, or PLGA@hdCVB3I4T1M) twice on days 0 and 7 (10 mg mL^−1^ PLGA). Body weight was recorded every 3 days. 18 days after the initial injection, the mice were sacrificed, and various tissues were collected for safety analysis using H&E staining. Additionally, blood samples were obtained to measure the levels of biochemical markers, including alanine aminotransferase (ALT), aspartate aminotransferase (AST), and creatinine.

### 2.12 Assessment of Tumor‐ and Virus‐Specific T Cell Responses Following PLGA@hdCVB3I4T1M Vaccination

To evaluate the antigen‐specific T cell responses induced by the PLGA@hdCVB3I4T1M vaccine and assess potential immune activation against tumor‐ versus virus‐derived components, ex vivo restimulation assays were performed using splenocytes from vaccinated mice. Tumor‐free Balb/c mice (*n* = 3 per group) were vaccinated subcutaneously on days 0 and 7 with PLGA@hdCVB3I4T1M. One week after the final immunization, spleens were collected and processed into single‐cell suspensions following mechanical disruption and red blood cell lysis. Splenocytes (2 × 10^5^ cells/well) were cultured in 24‐well round‐bottom plates and restimulated for 72 h with either i) 4T1 tumor cell lysate (to assess tumor‐specific responses), ii) hdCVB3 or iii) PBS (negative control). After incubation, culture supernatants were harvested, and levels of IFN‐γ and TNF‐α were quantified using commercial ELISA kits according to the manufacturer's instructions.

### 2.13 Visualization of Vaccine Accumulation in Lymph Nodes

To assess the biodistribution and uptake of the PLGA@hdCVB3I4T1M vaccine in lymphoid tissues, vaccinated and control (PBS‐injected) Balb/c mice were sacrificed 24 h post‐injection, and inguinal lymph nodes were harvested. The vaccine formulation was fluorescently labeled with Nile Red (2916406, Invitrogen) to enable tracking by confocal microscopy. Freshly collected lymph nodes were embedded in optimal cutting temperature compound, snap‐frozen in liquid nitrogen, and stored at −80 °C. Cryosections (8 µm thick) were prepared using a cryostat (Leica CM1950) and mounted on glass slides for immunofluorescent staining. Tissue sections were fixed with cold acetone for 10 min, air‐dried, and then blocked with 5% BSA in PBS for 1 h at room temperature. Sections were incubated overnight at 4 °C with an anti‐CD11c antibody conjugated to Alexa Fluor 488 (117311, BioLegend) to visualize dendritic cells. After washing with PBS, nuclei were counterstained with DAPI, and slides were mounted with anti‐fade mounting medium. Confocal images were acquired using a Zeiss LSM880 confocal microscope.

### 2.14 Therapeutic Effect and Immune Stimulatory Effect of Vaccines

Female Balb/c mice (6–8 weeks old) were subcutaneously injected with 3 × 10^5^ 4T1 cells into the right flank. Once tumors reached ≈50 mm^3^, the mice were randomly assigned into four groups (*n* = 6 per group) and administered one of the following treatments: sham (PBS), PLGA@hdCVB3, PLGA@I4T1M, or PLGA@hdCVB3I4T1M. Injections were given intratumorally on days 0 and 5 (10 mg mL^−1^, PLGA). Tumor size was measured every three days using a digital caliper, and the tumor volume (mm^3^) was calculated using the formula: width^2^ × length × 0.5. The tumor suppression rate (TSR) was determined as: TSR (%) = [1 – (tumor volume of the treated group)/(tumor volume of the control group)] × 100%. Mice were euthanized if they met any of the following criteria: a body weight loss of ≥20%, tumor ulceration covering ≥10% of the surface, tumor diameter ≥1.7 cm, or a tumor weight exceeding 10% of body weight. Survival rates for each group were also recorded to assess treatment efficacy.

To investigate the immune‐stimulatory effects of the treatments, tumors were harvested from a different cohort of four female Balb/c mice per group on day 14 post‐treatment, fixed in 4% paraformaldehyde, and processed for immunohistochemistry (IHC). Tumor sections underwent deparaffinization, rehydration, and staining with antibodies against CD8 (98941, Cell Signaling Technology), F4/80 (70076, Cell Signaling Technology), FoxP3 (12653, Cell Signaling Technology), and granzyme B (4275, Cell Signaling Technology). IHC was performed using the MACH4 Universal HRP‐Polymer Detection System (BRI4012H, Biocare Medical) and Gill II hematoxylin (GHS232, Sigma–Aldrich) according to established protocols. Digital images were captured using the Aperio ScanScope AT (Leica Biosystems Inc.), and quantitative analysis was conducted using NIH ImageJ software (version 1.52p), with results expressed as relative optical density.

Additionally, the levels of pro‐inflammatory cytokines, including IFN‐γ and TNF‐α in the tumor microenvironment as well as PD‐L1, were assessed via immunofluorescence staining using anti‐IFN‐γ (505802, BioLegend) and anti‐TNF‐α (506302, BioLegend) antibodies. Moreover, serum samples collected from treated mice were analyzed for levels of IFN‐γ, TNF‐α, and IL‐6 using ELISA kits (ab222503, Abcam) following the manufacturer's instructions.

### 2.15 Assessment of Tumor Preventive Properties

Female Balb/c mice (6–8 weeks old) were randomly assigned to four groups (*n* = 6 per group). Each group received subcutaneous vaccinations on days −14 and −7. On day 0, all mice were challenged with 1 × 10^6^4T1 cells injected into the right flank. The antitumor study was conducted as described above. In a separate study with the same procedure, on day 22 post‐challenge, tumors were harvested from four mice per group, fixed in 4% paraformaldehyde, and processed for IHC as described above. Additionally, pro‐inflammatory cytokines such as IFN‐γ and TNF‐α, in the tumor microenvironment, were assessed by immunofluorescence, as described above. The level of various cytokines in the serum of mice was also quantified using ELISA kits.

### 2.16 The Therapeutic Effect of Nanovaccine Combined with Oncolytic miR‐CVB3

A total of 5 × 10^5^ 4T1 cells were injected subcutaneously into the right flank of each mouse (female Balb/c mice, 6–8 weeks old). The next day, the mice were randomly divided into four groups (*n* = 6 per group) for different treatments: 1) sham (PBS), 2) PLGA@hdCVB3I4T1M, 3) miR‐CVB3, or 4) PLGA@hdCVB3I4T1M combined with miR‐CVB3. One day post‐tumor cell challenge (day 1), mice in groups 2 and 4 received subcutaneous vaccinations with PLGA@hdCVB3I4T1M (10 mg mL^−1^, PLGA), and on day 8, mice in groups 3 and 4 were given intratumoral injections of miR‐CVB3 at a dosage of 10^5^ plaque‐forming units (pfu) per mouse. The antitumor study was conducted as described above. In a separate study with the same procedure, on day 22 post‐challenge, tumors were harvested from four mice per group, fixed in 4% paraformaldehyde, and processed for IHC as described above. Additionally, pro‐inflammatory cytokines such as IFN‐γ and TNF‐α in the tumor microenvironment, along with PD‐L1 protein (detected using anti‐PD‐L1, A1645, ABclonal), were assessed by immunofluorescence, as described above. The level of various cytokines in the serum of mice was also quantified using ELISA kits. In addition, tumor tissues were harvested to assess apoptosis rates using the terminal deoxynucleotidyl transferase dUTP nick end labeling (TUNEL) assay following the manufacturer's instructions (G3250, Promega).

### 2.17 Statistical Analysis

Statistical evaluations were performed using GraphPad Prism software (version 8.0.1). Data are presented as the mean ± standard deviation (SD), with a minimum sample size of *n* ≥ 3 per group. Group comparisons were conducted using either an unpaired Student's *t*‐test or a one‐way ANOVA, followed by Tukey's post hoc test for multiple comparisons. Survival analysis was carried out using the log‐rank test. Statistical significance was defined as *p* < 0.05, with the following thresholds: *p* < 0.05 (^*^), *p* < 0.01 (^**^), *p* < 0.001 (^***^), and *p* < 0.0001 (^****^).

## 3 Results and Discussion

CVB3 has demonstrated significant antitumor activity both in vitro and in vivo by directly lysing cancer cells and inducing immunogenic cell death.^[^ ^30^ ^]^ This process leads to the release of tumor‐associated antigens, damage‐associated molecular patterns (DAMPs), and pathogen‐associated molecular patterns (PAMPs) into the tumor microenvironment, which in turn stimulates the immune system.^[^ ^30^ ^]^ Despite these promising effects, the clinical application of CVB3 has been hindered by off‐target toxicities, particularly pancreatotoxicity and cardiotoxicity.^[^ ^28^ ^]^

Our recent studies, along with findings from other groups, have revealed that CVB3 infection alters the expression of several proteins on cancer cells prior to cell lysis.^[^ ^31^ ^]^ This suggests that the surface proteins of infected cancer cells may play a key role in modulating immune responses. Therefore, in this study, we aimed to investigate the immunostimulatory proteins present on the membrane of CVB3‐infected cancer cells. By assessing the potential of these modified membranes, we sought to determine whether they may serve as a novel and effective platform for cancer vaccine development.

**Figure 1:**
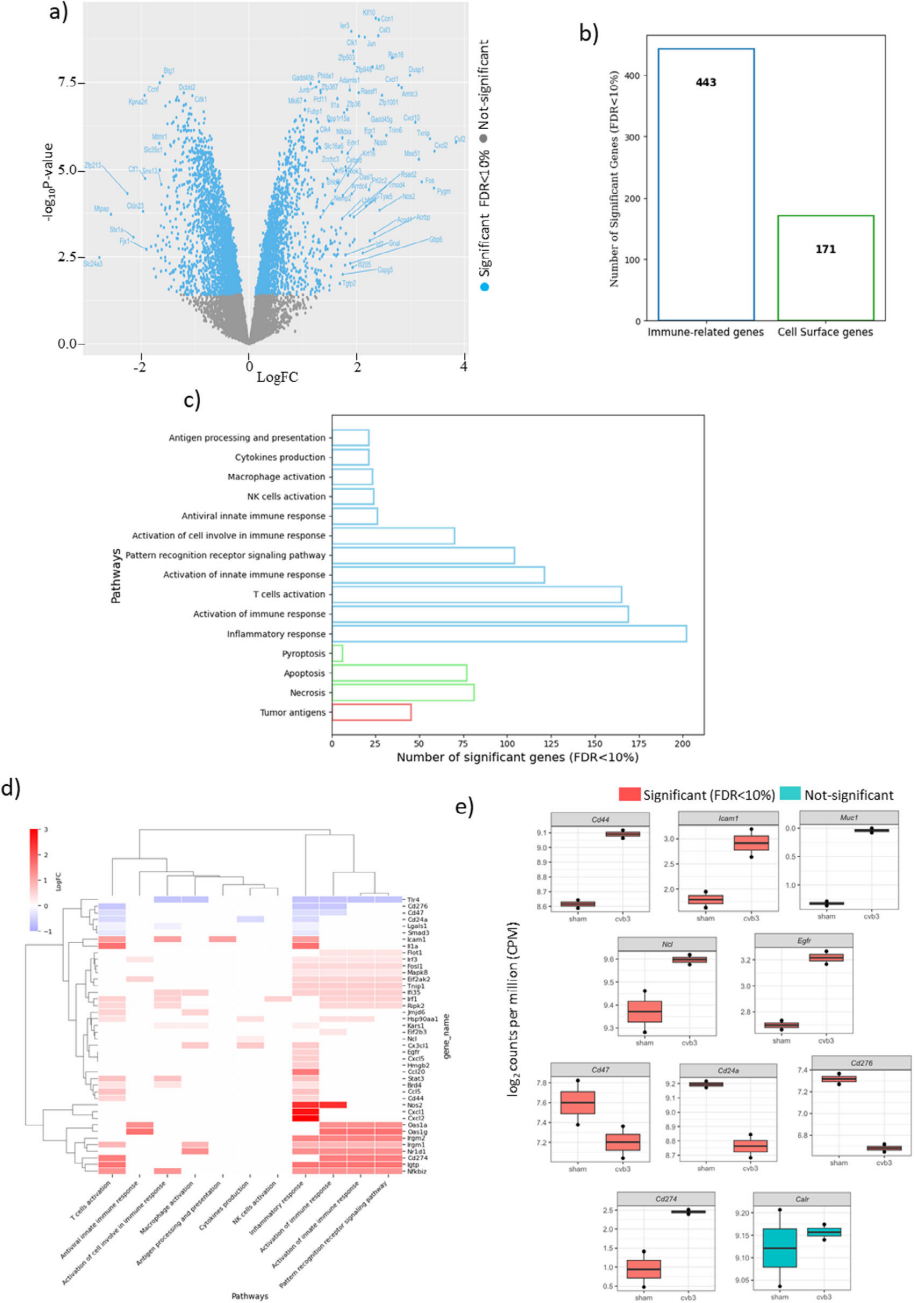
RNA sequencing analysis of cancer cells before and after CVB3 infection. a) Volcano plot showing differentially expressed genes following CVB3 infection, with significantly upregulated and downregulated genes highlighted. b) Bar charts depicting the number of significantly altered immune‐related genes and cell surface genes. c) Bar charts displaying the number of differentially expressed genes involved in key immune‐related pathways, including T cell activation, cytokine production, and antigen presentation, as well as cell death pathways such as pyroptosis and necrosis, and tumor antigen‐associated pathways. d) Heatmap illustrating the expression profiles of immune‐related genes and tumor antigens before and after infection. e) box plots of some selected tumor antigens and immune‐related genes post‐infection.

To investigate the transcriptional changes induced by CVB3 infection, we performed RNA‐seq on cancer cells before and after infection. Salmon indicated 95% alignment rates for sham samples and 85% alignment rates for CVB3‐infected samples. Interesting, the 10% was explained by aligning to the virus genome, where alignment rates for CVB3 were ≈10% to the CVB3 genome and 0% for sham samples. After removing low‐abundance transcripts, 11 309 transcripts were used to perform differential expression analysis (**Figure** **1**a). 4372 transcripts were differentially expressed (blue) where 1853 (2519) were up (down)‐regulated following infection (FDR<10%). To further characterize the immunological impact of CVB3 infection, we quantified the number of significantly altered genes associated with immune‐related functions and cell surface expression. Figure 1b demonstrates a substantial increase in differentially expressed immune‐related and cell surface genes following CVB3 infection. Next, we analyzed key immune‐related pathways affected by CVB3 infection. Our results revealed a significant increase in genes associated with cell death, tumor antigens, and immune pathways such as T cell activation, cytokine production, and antigen presentation, indicating potential enhancement of tumor immunogenicity (Figure 1c). To further illustrate the impact of CVB3 infection on immune‐related gene expression and tumor antigen, we generated a heatmap (Figure 1d) displaying the expression profiles of immune‐related genes and tumor antigens. This analysis revealed a distinct shift in gene expression patterns post‐infection, with upregulation of multiple immunostimulatory genes. Finally, Figure 1e presents box plots for selected tumor antigens and immune‐related genes, showcasing their expression profiles following CVB3 infection. These plots highlight the potential impact of the virus on reshaping cancer cell membrane, with some genes showing significant alterations indicative of immune activation. Together, these findings suggest that CVB3 infection modulates gene expression in a manner that enhances tumor immunogenicity, potentially improving the efficacy of immune‐based therapeutic strategies.

**Figure 2:**
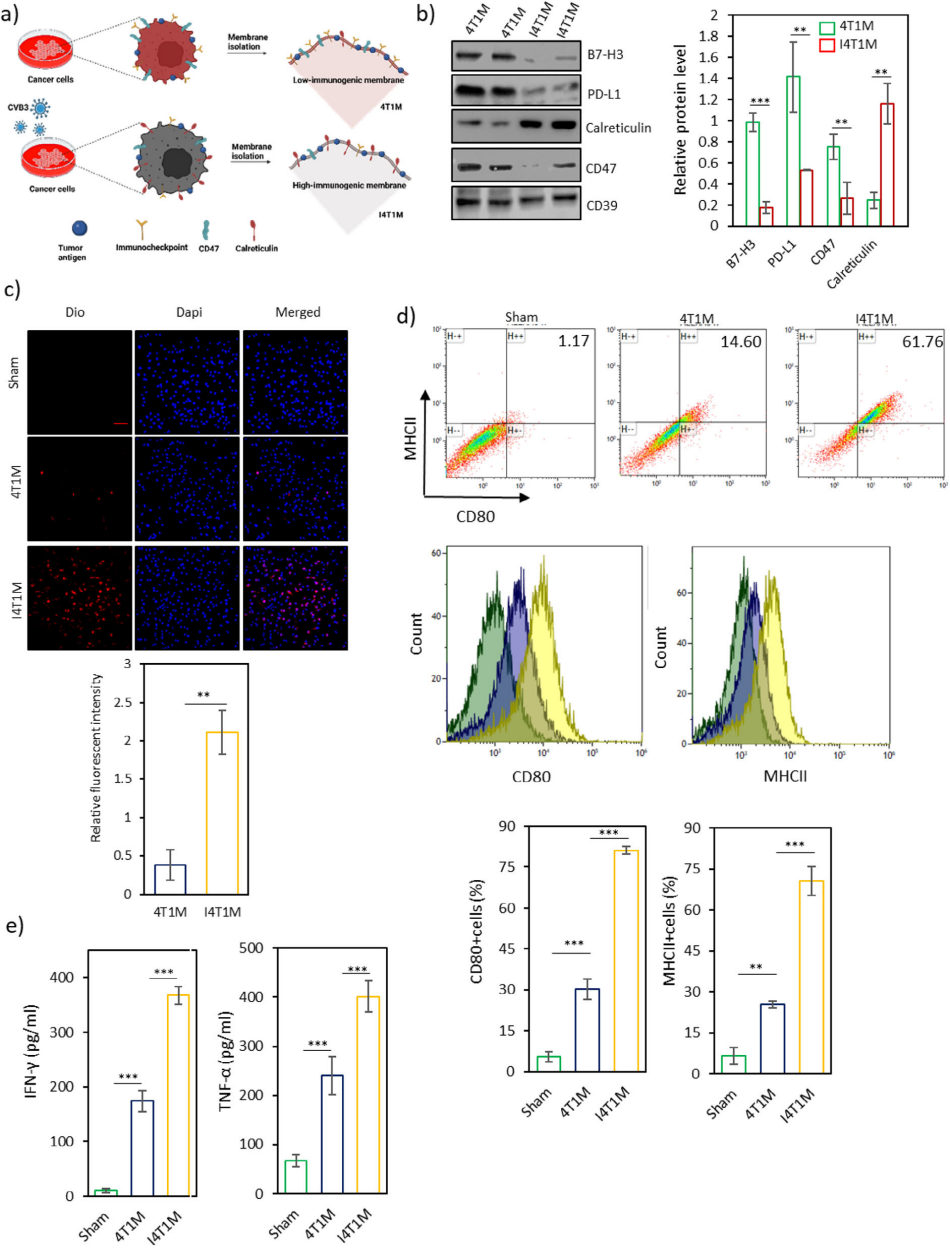
Characterization and immunostimulatory activity of membranes derived from non‐infected and CVB3‐infected 4T1 cancer cells. a) Schematic illustration of membrane isolation from non‐infected and CVB3‐infected 4T1 breast cancer cells (referred to as 4T1M and I4T1M, respectively). b) Western blot analysis of immune‐related proteins, B7‐H3, PD‐L1, Calreticulin, CD47, and CD39, in 4T1M and I4T1M. c) Confocal microscopy images showing the uptake of 4T1M and I4T1M by RAW264.7 macrophages after 2 h of incubation (scale bars: 50 µm), with corresponding fluorescence intensity quantification using ImageJ (version 1.52p) software. d) Flow cytometry analysis and quantification of maturation markers CD80 and MHCII on BMDCs after a 12 h incubation with 4T1M and I4T1M. e) Splenocytes from vaccinated mice were restimulated with 4T1 lysate, and ELISA showed significantly higher TNF‐α and IFN‐γ levels in the I4T1M group compared to 4T1M and PBS groups, indicating improved antigenicity. ^*^ *p* < 0.05; ^**^ *p* < 0.01; ^***^ *p* < 0.001; ^****^ *p* < 0.0001 by unpaired Student's *t*‐test or ANOVA.

Next, we isolated the membranes from 4T1 cancer cells before and after infection (referred to as 4T1M and I4T1M, respectively) for subsequent analysis (**Figure** **2**a). To evaluate the impact of CVB3 infection on immune‐related proteins, we directly analyzed some key proteins by western blot analysis (Figure 2b). Our results demonstrated that CVB3 infection led to a significant reduction in the expression of PD‐L1 and B7‐H3, two prominent immune checkpoint molecules that suppress immune cell activity by interacting with their respective receptors on immune cells (Figure 1b).^[^ ^32^, ^33^ ^]^ Interestingly, RNA sequencing analysis indicated an upregulation of PD‐L1 transcript levels following CVB3 infection (Figure 1e). However, western blot analysis revealed a decrease in PD‐L1 protein expression (Figure 2b). This suggests potential post‐translational regulation, such as protein degradation or modifications, which may influence PD‐L1 protein levels. Furthermore, the level of CD47, often referred to as the “don't eat me” signal, was significantly reduced in the membranes isolated from CVB3‐infected cancer cells, potentially enhancing immune recognition and reducing immune evasion (Figure 2b). In contrast, while RNA sequencing showed no significant increase in calreticulin expression at the transcript level, western blot analysis revealed a marked upregulation of calreticulin on the membranes of infected cancer cells (Figure 1b). This suggests that CVB3 infection may not directly influence calreticulin gene expression but instead promotes its translocation from the endoplasmic reticulum to the cell membrane, enhancing its recognition as an “eat me” signal for phagocytosis by antigen‐presenting cells (APCs).^[^ ^34^ ^]^ These findings highlight that CVB3 infection induces substantial alterations in the expression of immune‐related proteins on the membranes of cancer cells, shifting them toward a more immunostimulatory profile.

Next, we evaluated the cellular uptake and immunostimulatory properties of 4T1M and I4T1M. RAW 264.7 macrophages were treated with DiO‐labeled 4T1M and I4T1M. After a 2 h incubation, confocal microscopy revealed a significantly higher fluorescence intensity in cells treated with I4T1M, indicating enhanced phagocytosis (Figure 2c). This increase in uptake is likely attributed to alterations in immunostimulatory proteins on the infected membranes. Additionally, we evaluated the activation of RAW 264.7 cells and BMDCs by measuring the surface expression of CD80 and MHCII on macrophages and dendritic cells following treatment with each membrane type using flow cytometry. As shown in Figure 2d and Figure S1 (Supporting Information), the proportion of CD80^+^MHCII^+^ cells in the I4T1M group was more than three times higher than that in the 4T1M group. To further assess macrophage activation, we examined the induction of pro‐inflammatory cytokines such as *tnf‐α*, *cxcl10*, and *ccl5*. As shown in Figure S2 (Supporting Information), I4T1M demonstrated a significantly higher capacity to induce the gene expression of these cytokines compared to 4T1M. These findings suggest that the changes in immunostimulatory proteins on the cancer cell membranes following CVB3 infection, including a reduction in PD‐L1, B7‐H3, and CD47, and an increase in calreticulin, are more conducive to activating the anti‐tumor immune response.

To further examine whether CVB3 infection enhances the immunogenicity of tumor membranes and promotes tumor‐specific T cell responses, we performed an ex vivo restimulation assay using 4T1 tumor cell lysates. Splenocytes from mice vaccinated with either I4T1M or 4T1M were incubated with the lysates, and TNF‐α and IFN‐γ levels in the supernatants were measured. Both vaccinated groups showed elevated cytokine secretion compared to PBS controls, indicating successful induction of tumor‐reactive T cells. Notably, splenocytes from I4T1M‐vaccinated mice produced significantly higher levels of both cytokines than those from the 4T1M group, suggesting that CVB3 infection enhances tumor membranes immunogenicity and elicits a more potent tumor‐specific T cell response (Figure 2e).

After verifying the superior immunostimulatory properties of the membranes from CVB3‐infected cancer cells compared to non‐infected membranes, we aim to develop a personalized nanovaccine for breast cancer treatment. This approach utilizes a PLGA‐based nanoparticle platform, where the surface of the PLGA nanoparticle was coated with I4T1M, while the core was loaded with hdCVB3. Heat‐inactivated viruses have been shown to activate several immunological pathways in APCs, leading to enhanced immune activation. In addition to its immunostimulatory effects, the use of hdCVB3 also serves to prime the immune system against the virus. This strategy aims to enhance the efficacy of subsequent treatments with oncolytic CVB3 or its safer variant, miR‐CVB3, by leveraging an enhanced immune response. Although not directly assessed in this study, the risk of inducing strong neutralizing immunity is likely low due to the heat‐inactivated nature of the virus and its encapsulation within PLGA nanoparticles. By combining the nanovaccine with oncolytic miR‐CVB3, we anticipate a synergistic effect, resulting in a more robust antitumor immune response.

**Figure 3:**
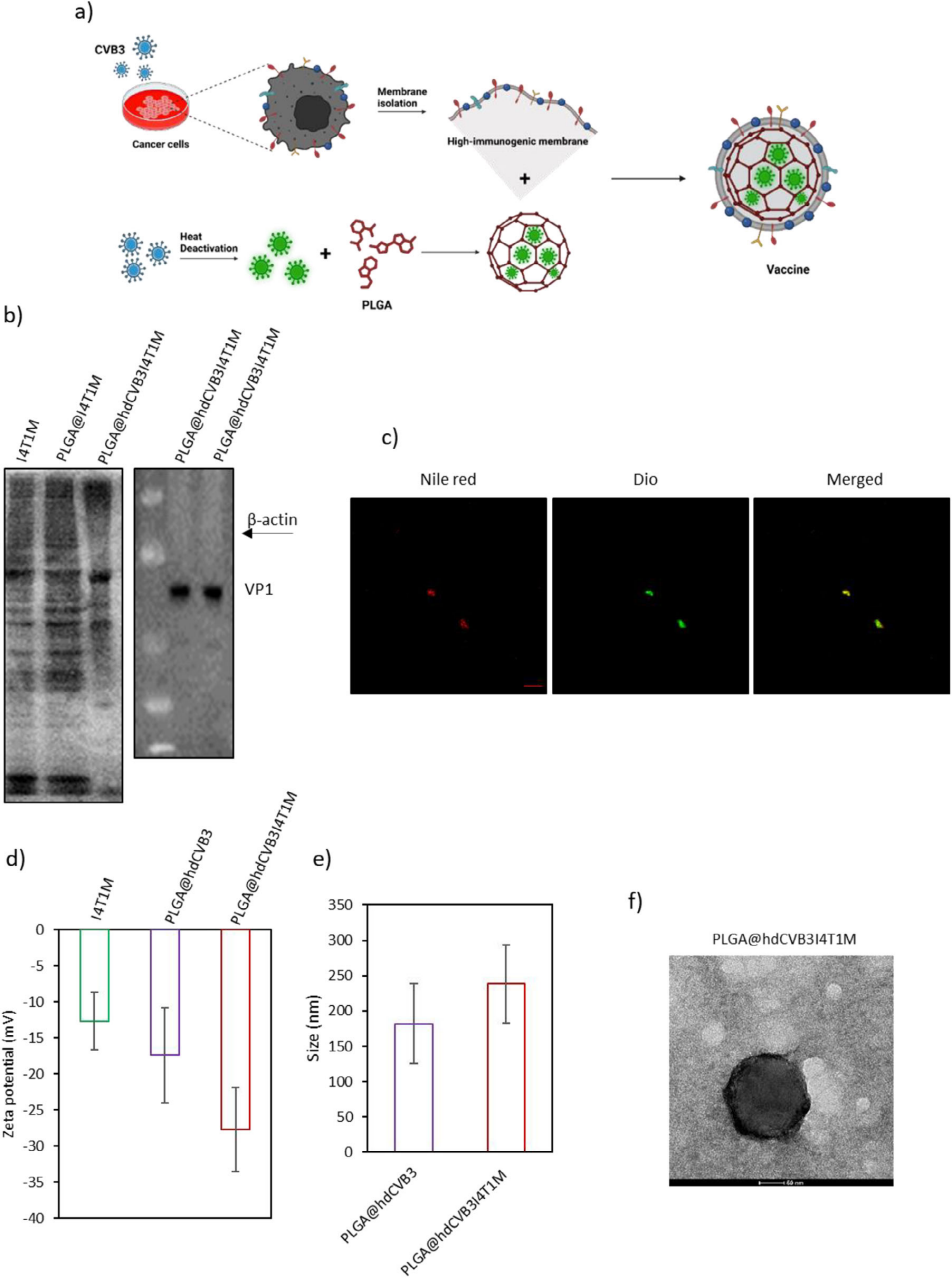
Synthesis and characterization of PLGA@hdCVB3I4T1M nanoparticles. a) Schematic representation of the preparation steps for PLGA@hdCVB3I4T1M nanoparticle formulation. b) SDS‐PAGE protein analysis comparing I4T1M, PLGA@I4T1M, and PLGA@hdCVB3I4T1M, along with western blot analysis of VP1 and β‐actin in the PLGA@hdCVB3I4T1M vaccine. c) Fluorescent microscopy images of PLGA@hdCVB3I4T1M labeled with DiO and Nile red dyes to visualize proper formation of vaccine (scale bars: 10 µm). d,e) Measurement of particle size and zeta potential across different vaccine formulations. f) TEM image showing the size and morphological characteristics of PLGA@hdCVB3I4T1M nanoparticles. ^*^ *p* < 0.05; ^**^ *p* < 0.01; ^***^ *p* < 0.001; ^****^ *p* < 0.0001 by unpaired Student's *t*‐test or ANOVA.

The synthesis process of the PLGA@hdCVB3I4T1M personalized vaccine is illustrated in **Figure** **3**a. 4T1 cells were infected with CVB3 at an MOI of 1. The following day, after observing morphological changes indicative of cell death, the infected cells were harvested, and their membranes were isolated as described above. Concurrently, hdCVB3, serving as a PAMP, was encapsulated into PLGA nanoparticles (PLGA@hdCVB3). To create the PLGA@hdCVB3I4T1M personalized vaccine, equal amounts of I4T1M and PLGA@hdCVB3 nanoparticles were mixed and magnetically stirred overnight at 4 °C. To confirm the successful loading of hdCVB3 into the PLGA core and the coating of I4T1M on the surface, we performed SDS‐PAGE. As shown in Figure 3b, the protein bands for I4T1M, PLGA@I4T1M, and PLGA@hdCVB3I4T1M were consistent, indicating successful membrane coating. Additionally, the absence of β‐actin staining in PLGA@hdCVB3I4T1M group confirmed the absence of internal cellular proteins in the vaccine formulation (Figure 3b). Furthermore, staining for the viral capsid protein VP1 showed the presence of viral particles inside the PLGA nanoparticles, verifying proper encapsulation (Figure 3b). For further confirmation of successful vaccine synthesis, Nile Red and DiO were used to label the PLGA core and the I4T1M, respectively. Fluorescent imaging of PLGA@hdCVB3I4T1M revealed that the red fluorescence from the PLGA core and the green fluorescence from the cell membrane overlapped to form a yellow signal, confirming that the cell membrane was successfully coated on the surface of the PLGA nanoparticles (Figure 3c).

DLS and TEM were employed to analyze the particle size and microstructure of the personalized vaccine. DLS measurements showed that the zeta potential of I4T1M and PLGA@hdCVB3 were −12.7 ± 3.99 and −17.4 ± 6.62 mV, respectively, whereas PLGA@hdCVB3I4T1M exhibited a more negative zeta potential of −27.7 ± 5.84 mV (Figure 3d), suggesting successful membrane coating. PLGA@hdCVB3 exhibited an average diameter of 182 ± 56.35 nm, which increased to 238.1 ± 55.21 nm after the addition of the I4T1M membranes, further confirming the successful coating process (Figure 3e). TEM imaging provided additional verification, revealing a distinct core–shell structure in PLGA@hdCVB3I4T1M, indicating successful incorporation of the I4T1M membrane onto the PLGA@hdCVB3 nanoparticles (Figure 3f).

**Figure 4:**
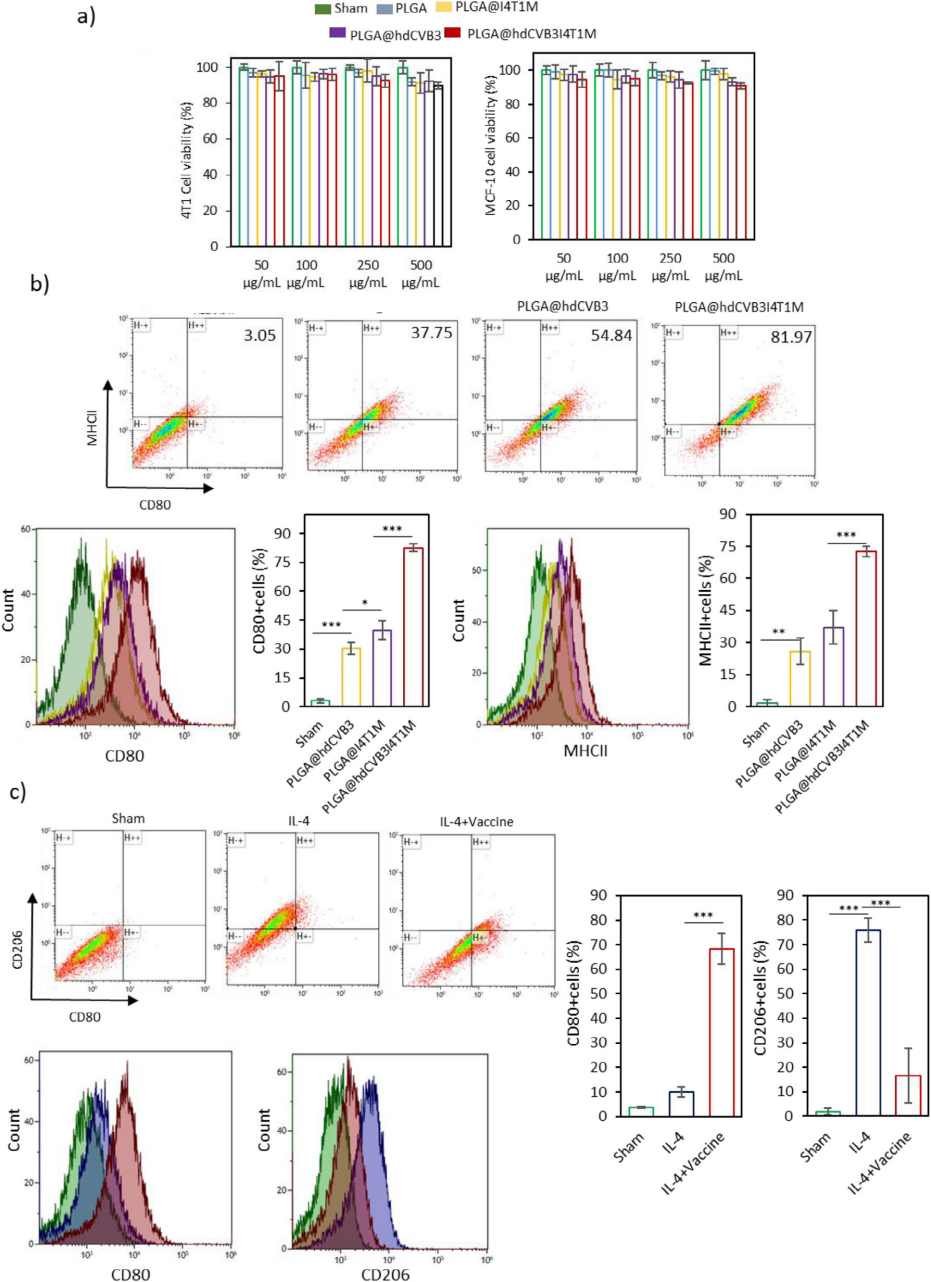
In vitro assessments of cell viability and immune cells stimulation. a) Cell viability analysis of 4T1 cancer cells and non‐tumorigenic human mammary epithelial MCF‐10A cells treated with different vaccine formulations as indicated for 36 h. b) Flow cytometry analysis of the maturation markers CD80 and MHCII on BMDCs after 12 h incubation with PLGA@I4T1M, PLGA@hdCVB3, and PLGA@hdCVB3I4T1M, with corresponding quantitative data. c) RAW 264.7 cells were polarized with IL‐4, then treated with PBS or PLGA@hdCVB3I4T1M for 24 h. Cells were stained for CD80 (M1) and CD206 (M2) and analyzed by flow cytometry to determine repolarization. ^*^ *p* < 0.05; ^**^ *p* < 0.01; ^***^ *p* < 0.001; ^****^ *p* < 0.0001 by ANOVA.

Assessing the cytotoxicity of nanomaterials is essential for their biological application. To evaluate the safety of the developed vaccines, various concentrations (0–500 µg mL^−1^) of PLGA@hdCVB3I4T1M, along with other formulations of vaccines, were co‐cultured with 4T1 and MCF‐10A cells, and cell viability was measured using the MTS assay. As shown in **Figure** **4**a, no significant cytotoxicity was observed in either cell line, even at the highest concentration of 500 µg mL^−1^, indicating the favorable safety profile of vaccines. Next, we investigated the immune‐stimulating potential of PLGA@hdCVB3I4T1M. First, the capacity of the different vaccine formulations to promote BMDCs and macrophage maturation was assessed by flow cytometry, using CD80 and MHCII as surface markers. While both PLGA@I4T1M and PLGA@hdCVB3 stimulated BMDCs and macrophage activation, PLGA@hdCVB3I4T1M induced significantly stronger maturation, further validating its superior potential for immune cell stimulation and activation (Figure 4b; Figure S3, Supporting Information). We also evaluated the induction of pro‐inflammatory cytokines in RAW 264.7 macrophages following treatment with various formulations of the nanovaccine. RT‐qPCR analysis revealed that both PLGA@hdCVB3 and PLGA@I4T1M significantly induced the production of pro‐inflammatory cytokines, including *tnfα*, *ccl5*, and *ifnb1* (Figure S4, Supporting Information). However, the highest cytokine levels were observed in the group treated with PLGA@hdCVB3I4T1M, indicating a synergistic effect of PAMPs and the infected membrane in stimulating immune cells (Figure S4, Supporting Information). Furthermore, western blot analysis of COX‐2, a marker of macrophage activation,^[^ ^35^ ^]^ showed the highest expression in the PLGA@hdCVB3I4T1M group, further confirming its potent ability to activate macrophages (Figure S5, Supporting Information). To evaluate the effect of the PLGA@hdCVB3I4T1M vaccine on macrophage polarization, RAW 264.7 cells were first polarized to an M2‐like state using IL‐4 and then treated with either PBS or the vaccine. Flow cytometric analysis revealed that vaccine‐treated macrophages exhibited a marked increase in CD80 expression and a concurrent reduction in CD206 expression compared to the PBS controls. These results indicate that PLGA@hdCVB3I4T1M effectively repolarizes M2‐like macrophages toward a pro‐inflammatory M1 phenotype, suggesting its potential to reprogram the immunosuppressive tumor microenvironment (Figure 4c). To assess the antigen specificity of T cell responses induced by PLGA@hdCVB3I4T1M, splenocytes from vaccinated, tumor‐free mice were restimulated ex vivo with either 4T1 tumor cell lysate or hdCVB3. ELISA analysis revealed that both stimuli significantly increased secretion of IFN‐γ and TNF‐α compared to PBS‐treated controls, indicating the presence of vaccine‐primed, antigen‐responsive T cells. Notably, stimulation with 4T1 tumor lysate elicited significantly higher cytokine production than stimulation with hdCVB3, suggesting a more robust T cell response directed against tumor‐associated antigens (Figure S6, Supporting Information).

**Figure 5:**
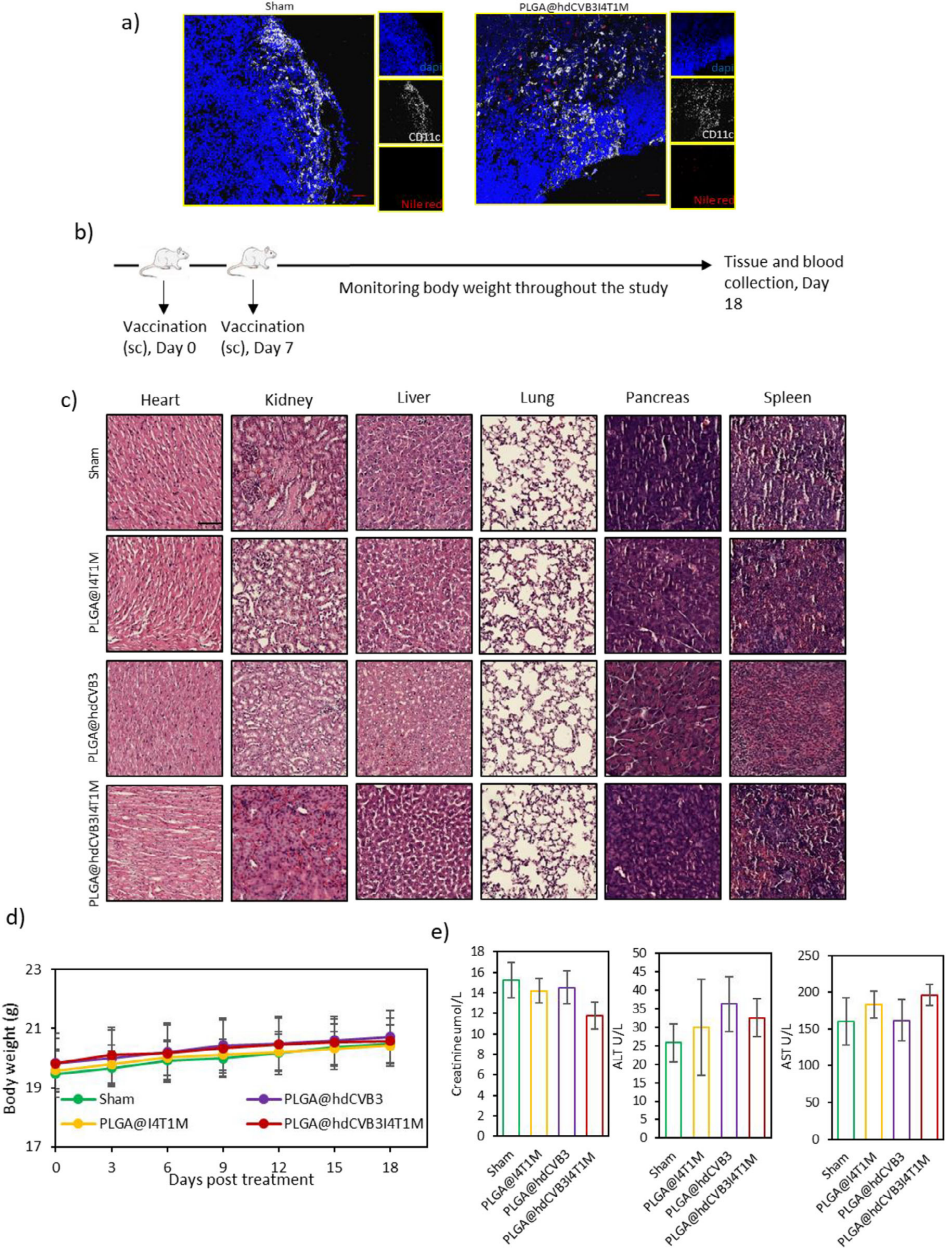
In vivo lymph node targeting and safety analysis of the developed vaccines. a) Confocal images of lymph node sections 24 h after injection of Nile Red‐labeled PLGA@hdCVB3I4T1M or PBS. Vaccine particles (red) accumulated in vaccinated mice and partially co‐localized with CD11c⁺ dendritic cells (white), indicating effective lymphatic targeting (scale bars: 50 µm). b) Schematic diagram outlining the safety analysis in mice (*n* = 5). c) Histopathological analysis of major organs (heart, kidney, liver, lung, pancreas and spleen) from mice at 18 days post‐administration of various vaccine formulations by H&E staining (scale bars: 100 µm). d) Monitoring of mouse body weight over 18 days following administration of different vaccine formulations to assess systemic toxicity. e) Assessment of serum biochemical markers, including creatinine, alanine aminotransferase (ALT), and aspartate aminotransferase (AST), at 18 days post‐vaccination. ^*^ *p* < 0.05; ^**^ *p* < 0.01; ^***^ *p* < 0.001; ^****^ *p* < 0.0001 by unpaired Student's *t*‐test or ANOVA.

To evaluate lymph node targeting and uptake of the PLGA@hdCVB3I4T1M vaccine, inguinal lymph nodes were collected from vaccinated and PBS‐treated mice 24 h post‐injection and analyzed by confocal microscopy. Nile Red fluorescence revealed strong accumulation of the vaccine in the lymph nodes of vaccinated mice, whereas no signal was observed in PBS‐treated controls. Co‐staining with the dendritic cell marker CD11c demonstrated spatial proximity and partial co‐localization of the vaccine with CD11c⁺ cells, indicating efficient uptake or interaction of the vaccine with resident dendritic cells in the draining lymph nodes (**Figure** **5**a). These findings confirm successful lymphatic trafficking of the PLGA@hdCVB3I4T1M formulation and suggest its potential to facilitate antigen presentation and initiate immune priming.

Next, we assessed the biosafety of the various vaccine formulations in Balb/c mice. After administering two doses of the vaccine (on days 0 and 7), multiple tissues, including the heart, liver, spleen, lungs, kidneys, and pancreas, were collected on day 18 for safety evaluation (Figure 5b). Histological analysis using H&E staining revealed no significant morphological changes, with all tissues displaying normal structure and no signs of toxicity (Figure 5c). Furthermore, no significant changes in body weight were observed throughout the monitoring period across the treatment groups (Figure 5d). In addition, blood analysis was performed to assess systemic biosafety. As shown in Figure 5e, serum levels of ALT, AST, and creatinine were not significantly different between the groups, and all values remained within the normal reference range. These findings demonstrate that the developed personalized nanovaccine exhibits excellent biological safety in vivo.

**Figure 6:**
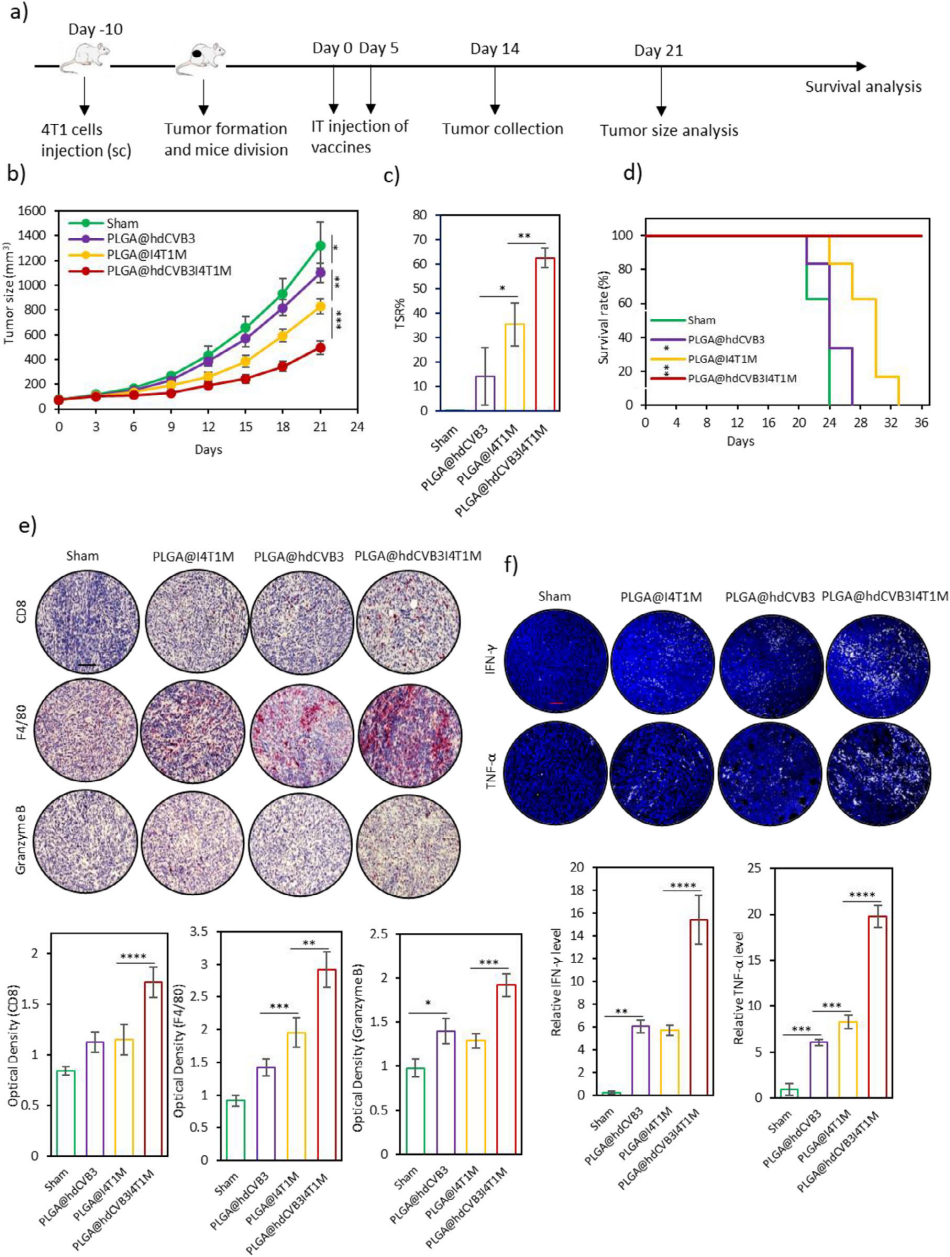
In vivo antitumor activity of various vaccines. a) Schematic depiction of the experimental design, illustrating the establishment of the 4T1 tumor‐bearing mouse model and subsequent treatment regimen. b) Tumor volume progression curves (*n* = 6 per group) following administration of different treatments. c) Tumor suppression rates across treatment groups on day 21. d) Survival rates of mice post‐treatment with different vaccines, showing long‐term therapeutic outcomes. e) Immunohistochemistry analysis of CD8+ T cells, F4/80 macrophages, and secreted granzyme B in tumor tissues from each treatment group (scale bars: 100 µm), with corresponding quantitative data (*n* = 4). f) Immunofluorescence staining of pro‐inflammatory cytokines IFN‐γ and TNF‐α within tumor tissues after each treatment (scale bars: 50 µm), with relative quantification of cytokine expression (*n* = 4). ^*^ *p* < 0.05; ^**^ *p* < 0.01; ^***^ *p* < 0.001; ^****^ *p* < 0.0001 by ANOVA.

Following that, we assessed the therapeutic efficacy of the developed vaccines in a 4T1 tumor‐bearing mouse model. Mice were divided into four groups: 1) PBS (Sham group), 2) PLGA@hdCVB3, 3) PLGA@I4T1M, and 4) PLGA@hdCVB3I4T1M. Each group received two intratumoral injections on days 0 and 5 (**Figure** **6**a). Tumor growth analysis showed that the PLGA@hdCVB3 group exhibited a slight reduction in tumor size, with an average size of 1099.46 mm^3^ compared to 1322.84 mm^3^ in the sham group (Figure 6b). This reduction is likely attributable to nonspecific immune stimulation by PAMPs within the tumor microenvironment. In the PLGA@I4T1M group, tumor suppression was more substantial, compared to both the control and PLGA@hdCVB3 groups (Figure 6b,c). Notably, the PLGA@hdCVB3I4T1M group showed the most significant inhibition of tumor growth, achieving a TSR of 62.47%. This finding underscores the enhanced therapeutic potential of combining infected membranes with hdCVB3 as a PAMP (Figure 6b,c). We further demonstrated that administration of PLGA@I4T1M improved survival rates compared to the other groups (Figure 6d). Remarkably, mice treated with PLGA@hdCVB3I4T1M showed the highest survival rates, with all animals surviving up to 36 days post‐treatment—significantly longer than those in other groups (Figure 6d).

To further understand the superior antitumor effect of PLGA@hdCVB3I4T1M, we analyzed immune cell infiltration in tumor tissues, focusing on cytotoxic T‐cell subpopulations, regulatory T cells, macrophages, and levels of pro‐inflammatory cytokines (TNF‐α, IFN‐γ) and granzyme B. Immune cell infiltration, specifically of CD8^+^ T‐cells and F4/80 macrophages, was evaluated using IHC staining. The PLGA@hdCVB3 group demonstrated a modest increase in CD8^+^ T cell and macrophage infiltration compared to the control group (Figure 6e). The PLGA@I4T1M group induced a more pronounced increase in immune cell infiltration, whereas the PLGA@hdCVB3I4T1M group elicited the most robust response, with over a 2‐fold increase in CD8^+^ T cells and a 3‐fold increase in macrophages compared to the sham group (Figure 6e). In addition, FoxP3 IHC staining revealed a noticeable reduction in regulatory T cell infiltration in the PLGA@hdCVB3I4T1M group compared to other groups, suggesting a shift toward a more immunostimulatory tumor microenvironment (Figure S7, Supporting Information). Furthermore, granzyme B levels were significantly elevated in the PLGA@hdCVB3I4T1M group, reinforcing its enhanced immune activation potential (Figure 6e). These results suggest that the PLGA@hdCVB3I4T1M vaccine exerts a potent antitumor effect by promoting vigorous immune cell infiltration into the tumor microenvironment, thereby enhancing the antitumor immune response.

Moreover, immunofluorescent staining revealed increased production of TNF‐α and IFN‐γ across all treatment groups (Figure 6f). Specifically, both the PLGA@hdCVB3 and PLGA@I4T1M groups showed significant increases in these pro‐inflammatory cytokines, with the highest expression observed in the PLGA@hdCVB3I4T1M group, which significantly surpassed all other groups (Figure 6f). To further assess systemic immune activation, serum cytokine levels were measured by ELISA. Consistent with the local immune activation, circulating TNF‐α, IFN‐γ, and IL‐6 levels were significantly elevated in the PLGA@hdCVB3I4T1M group compared to the controls and other treatment groups (Figure S8, Supporting Information), further supporting the systemic immunostimulatory effects of the vaccine.

**Figure 7:**
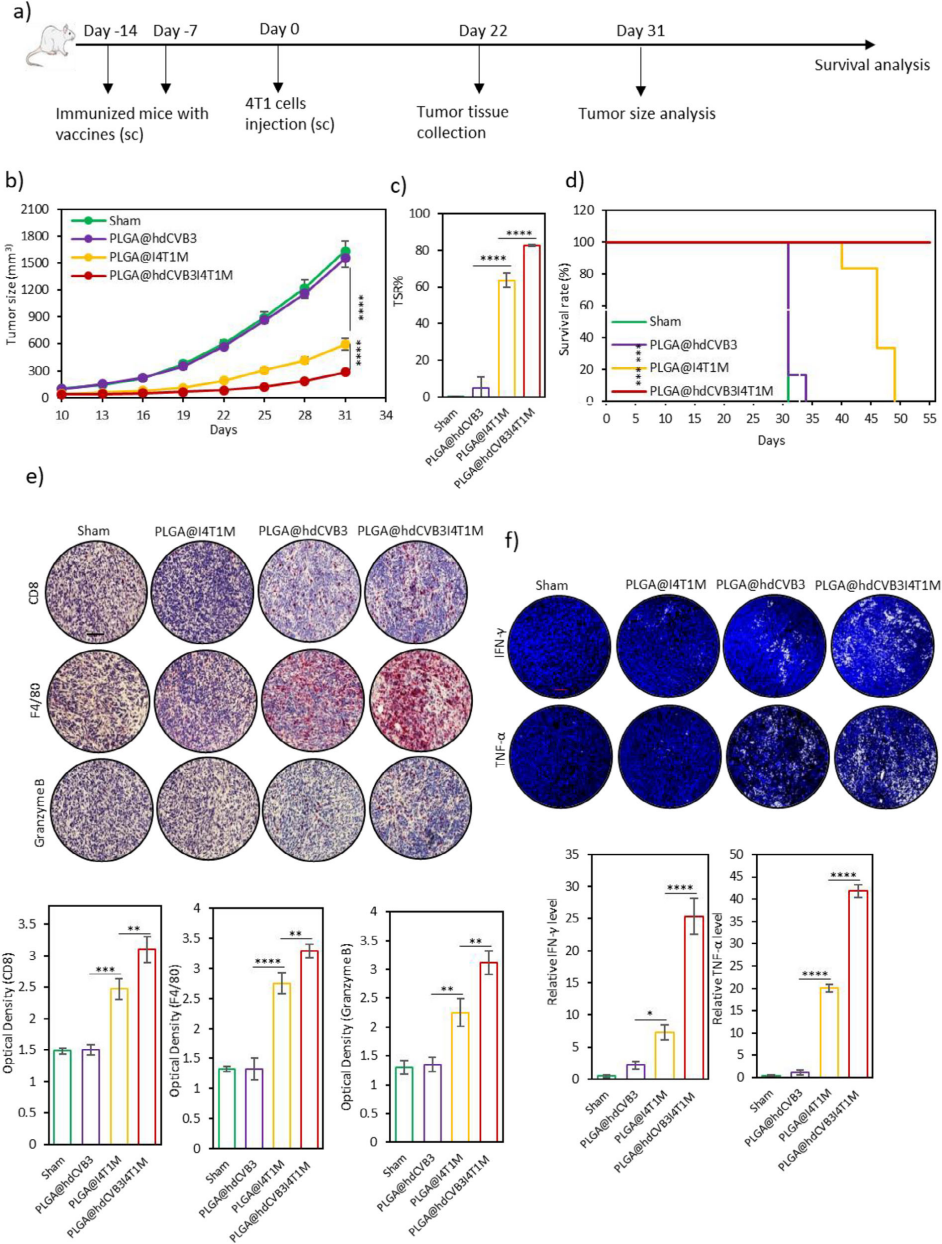
Prophylactic effects of different nanovaccine formulations. a) Schematic diagram outlining the vaccination schedule and subsequent 4T1 tumor challenge in mice. b) Average tumor growth curves in mice from different groups (*n* = 6 per group) following various vaccination regimens and 4T1 tumor challenge. c) Tumor suppression rates observed in mice after receiving different vaccine formulations on day 31. d) Survival curves of 4T1 tumor‐bearing mice across different vaccination groups, demonstrating long‐term protective effects (The sharp drop in the Sham group reflects euthanasia at predefined humane endpoints, not natural death). e) Immunohistochemistry analysis of CD8+ T cells, F4/80 macrophages, and secreted granzyme B in tumor tissues (scale bars: 100 µm), with corresponding quantitative assessment on day 22 (*n* = 4). f) Immunofluorescence staining of pro‐inflammatory cytokines IFN‐γ and TNF‐α in tumors following each vaccination (scale bars: 50 µm), with relative quantification of cytokine expression on day 22 (*n* = 4). ^*^ *p* < 0.05; ^**^ *p* < 0.01; ^***^ *p* < 0.001; ^****^ *p* < 0.0001 by ANOVA.

Next, we evaluated the prophylactic effects of the developed nanovaccines in vivo in the 4T1 tumor model. As shown in **Figure** **7**a, mice were immunized subcutaneously with different vaccine formulations: PLGA@hdCVB3, PLGA@I4T1M, or PLGA@hdCVB3I4T1M. Each vaccine was administered twice at one‐week intervals. Seven days after the final immunization, the mice were challenged with 4T1 cells (Day 0), and tumor growth was monitored starting from day 10 post‐tumor inoculation.

As anticipated, no significant difference in tumor growth was observed between the PLGA@hdCVB3 group and the sham group (Figure 7b,c). In contrast, vaccination with PLGA@I4T1M significantly suppressed tumor growth compared to the sham group. By the end of the antitumor study, the average tumor size in the PLGA@I4T1M group was 594.68 mm^3^ (with a TSR of 67.25%), significantly smaller than the average tumor sizes observed in the PLGA@hdCVB3 group (1553.5 mm^3^) and the sham group (1631.85.5 mm^3^, Figure 7b,c). The most profound tumor suppression was observed in mice immunized with PLGA@hdCVB3I4T1M, where the average tumor size reached only 283.06 mm^3^, with a TSR of 82.8%, demonstrating the strong antitumor efficacy of this formulation (Figure 7b,c). As expected, administration of PLGA@I4T1M significantly improved survival rates compared to the other groups (Figure 7d). Remarkably, mice treated with PLGA@hdCVB3I4T1M showed the highest survival rates, with all animals surviving up to 54 days post‐tumor challenge.

To further understand the immune mechanisms behind the antitumor effects, tumor tissues were harvested 22 days post‐tumor challenge for analysis of immune cell infiltration and proinflammatory cytokine production. Vaccination with PLGA@I4T1M significantly increased the infiltration of T cells and macrophages into the tumor microenvironment, along with elevated levels of TNF‐α, IFN‐γ, and granzyme B, compared to the sham and PLGA@hdCVB3 groups (Figure 7e,f). However, the most pronounced immune cell infiltration and cytokine production were observed in mice that received PLGA@hdCVB3I4T1M (Figure 7e,f). In addition, FoxP3 IHC staining revealed a significant reduction in regulatory T cell infiltration in the PLGA@hdCVB3I4T1M group, suggesting a decrease in immunosuppressive activity within the tumor microenvironment (Figure S9, Supporting Information). Consistent with these local immune responses, serum levels of TNF‐α, IFN‐γ, and IL‐6 were also significantly elevated in this group (Figure S10, Supporting Information), indicating robust systemic immune activation. These findings strongly support the efficacy of our nanovaccine design, which combines infected cancer cell membranes—harboring tumor‐specific antigens with reduced immunosuppressive proteins and enhanced immunostimulatory markers—and hdCVB3 as a potent adjuvant. This synergistic combination significantly enhances the immunogenicity of the vaccine, leading to strong antitumor immunity and improved survival in tumor‐bearing mice.

One of the key advantages of the developed personalized nanovaccine is the incorporation of hdCVB3 as a PAMP to serve as an adjuvant. This innovative approach enables the combination of cancer immunotherapy with oncolytic virotherapy. Since the immune system is primed against both CVB3 and tumor antigens upon vaccination, we hypothesized that subsequent treatment with CVB3 as an oncolytic virus will elicit a more robust immune response, thereby enhancing therapeutic efficacy.

**Figure 8:**
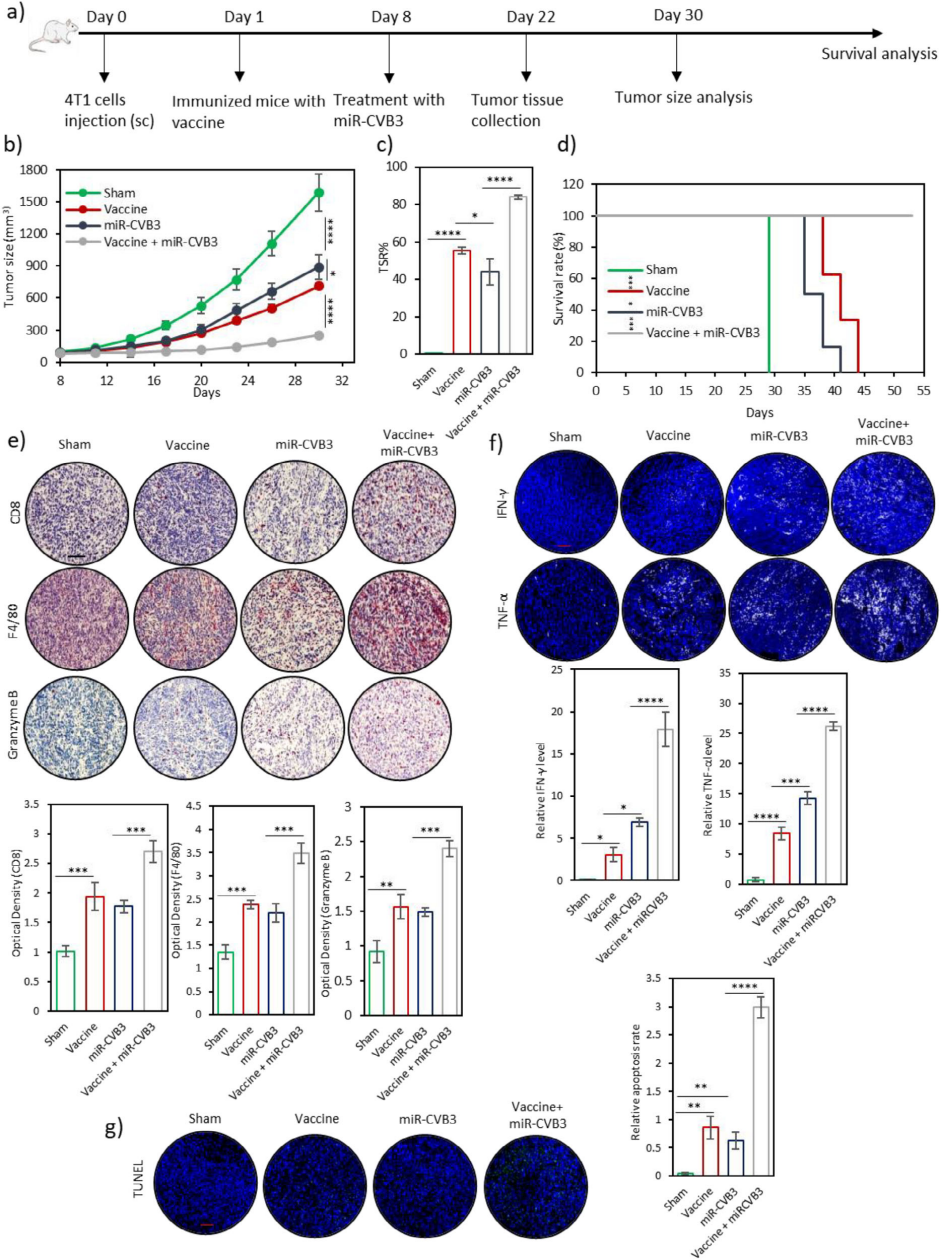
Antitumor effect of vaccination in combination with oncolytic virotherapy. a) Schematic illustration of the experimental design for evaluating the combination of vaccination and oncolytic virotherapy in a tumor‐bearing mouse model. b) Tumor growth profiles (*n* = 6 per group) in mice receiving different treatments. c) Tumor suppression rates in various treatment groups on day 30 post‐tumor challenge. d) Survival rates of mice subjected to different treatments (The sharp drop in the Sham group reflects euthanasia at predefined humane endpoints, not natural death). e) Immunohistochemistry analysis of CD8+ T cells, F4/80 macrophages, and secreted granzyme B in tumor tissues from each treatment group (scale bars: 100 µm), with corresponding quantification on day 22 (*n* = 4). f) Immunofluorescence staining of pro‐inflammatory cytokines IFN‐γ and TNF‐α in tumor regions after each treatment (scale bars: 50 µm), with relative quantification of cytokine expression on day 22 (*n* = 4). g) Terminal deoxynucleotidyl transferase dUTP nick‐end labeling (TUNEL) assay of tumor tissues (*n* = 4), showing apoptotic cell death (scale bars: 50 µm). ^*^ *p* < 0.05; ^**^ *p* < 0.01; ^***^ *p* < 0.001; ^****^ *p* < 0.0001 by ANOVA.

We previously engineered a safer version of CVB3 (miR‐CVB3) with proven antitumor activity.^[^ ^15^ ^]^ We next sought to evaluate whether combining vaccination with oncolytic virotherapy could improve therapeutic outcomes and survival rates in the 4T1 mouse tumor model. The experimental design is illustrated in **Figure** **8**a. On day 0, mice were challenged with 4T1 cells in the right flank. On day 1, mice in group 1 (Sham) received PBS, while those in groups 2 (PLGA@hdCVB3I4T1M) and 4 (PLGA@hdCVB3I4T1M combined with miR‐CVB3) were vaccinated with PLGA@hdCVB3I4T1M. On day 8, mice in groups 3 (miR‐CVB3 group) and 4 received an intratumoral injection of miR‐CVB3 (10^5^ pfu). Tumor‐bearing mice treated with only PLGA@hdCVB3I4T1M (group 2) or only miR‐CVB3 (group 3) exhibited comparable tumor growth rates and median survival. However, in stark contrast, mice receiving the combination of vaccination and oncolytic virotherapy exhibited significantly reduced tumor growth and TSR compared to both monotherapies. Moreover, this group demonstrated a markedly higher survival rate than either single‐treatment group (Figure 8b–d).

Immunological analyses revealed that the combination therapy markedly enhanced immune cell infiltration into the tumor microenvironment and resulted in the highest levels of proinflammatory cytokines and granzyme B expression (Figure 8e,f), along with significantly elevated serum concentrations of TNF‐α, IFN‐γ, and IL‐6 (Figure S11, Supporting Information). FoxP3 IHC further showed a substantial reduction in regulatory T cell infiltration in tumors following combination therapy, indicating a shift toward a more immunostimulatory tumor microenvironment (Figure S12, Supporting Information). In addition, immunofluorescent staining of tumor sections demonstrated reduced PD‐L1 expression in the PLGA@hdCVB3I4T1M + miR‐CVB3 group compared to monotherapy and control groups, suggesting decreased immune checkpoint signaling (Figure S13, Supporting Information). Furthermore, TUNEL staining showed a large number of apoptotic tumor cells in the PLGA@hdCVB3I4T1M + miR‐CVB3 group, while fewer apoptotic cells were observed in the monotherapy groups (Figure 8g). In contrast, almost no apoptotic cells were detected in the PBS group (Figure 8g). Taken together, these findings strongly suggest that the combination of the developed personalized cancer nanovaccine and oncolytic virotherapy synergistically enhances therapeutic efficacy and improves survival rates, offering a promising strategy for cancer treatment.

## 4 Conclusion

In this study, we developed and characterized a personalized nanovaccine using a combination of hdCVB3 and membranes isolated from infected 4T1 cells. The nanovaccine, PLGA@hdCVB3I4T1M, was successfully formulated with optimal biophysical properties and exhibited minimal cytotoxicity in both normal and cancer cells, highlighting its safety for in vivo applications. The unique design of this vaccine provides significant advantages. First, the use of infected cancer cell membranes ensures the presence of fewer immunosuppressive proteins and a higher concentration of immunostimulatory proteins. This combination improves immune system activation, making the vaccine more effective in stimulating an immune response. Second, the hdCVB3 acts as an adjuvant, activating multiple immune pathways, and further enhancing the vaccine's ability to elicit a vigorous antitumor response.

Our in vitro and in vivo evaluations demonstrated the strong immunostimulatory potential of PLGA@hdCVB3I4T1M. It significantly upregulated proinflammatory cytokines, such as TNF‐α and IFN‐γ, and promoted the activation of macrophages. Additionally, the vaccine enhanced T‐cell and macrophage infiltration into the tumor microenvironment, accompanied by increased granzyme B levels, which likely contributed to its potent antitumor effects. In prophylactic studies, the vaccine demonstrated remarkable tumor suppression, with the most significant delay in tumor growth and the highest levels of immune cell infiltration and cytokine production among the treatment groups. Moreover, combining this personalized nanovaccine with miR‐CVB3‐based oncolytic virotherapy synergistically improved therapeutic efficacy. This combination resulted in enhanced tumor cell apoptosis and prolonged survival rates in tumor‐bearing mice. Overall, these findings suggest that PLGA@hdCVB3I4T1M represents a promising and highly effective cancer immunotherapy approach. By integrating tumor‐specific antigens from infected cancer cell membranes and leveraging the immunostimulatory properties of hdCVB3, the vaccine not only activates immune cells through diverse pathways but also shows potential for combination with oncolytic virotherapy, offering a powerful and versatile tool for future cancer treatments.

## Conflict of Interest

The authors declare no conflict of interest.

## Supporting information



Supporting Information

## Data Availability

The data that support the findings of this study are available from the corresponding author upon reasonable request.
