# Supplementary material for: Developing a Personalized Cancer Nanovaccine Using Coxsackievirus‐Reprogrammed Cancer Cell Membranes for Enhanced Anti‐Tumor Immunity
Source: Adv Sci (Weinh). 2025 Jul 29;12(40):e06791. doi: 10.1002/advs.202506791 (PMC12561386; doi:10.1002/advs.202506791)
Supplement: Supplementary file 1 — Supporting Information [file ADVS-12-e06791-s001.pdf]

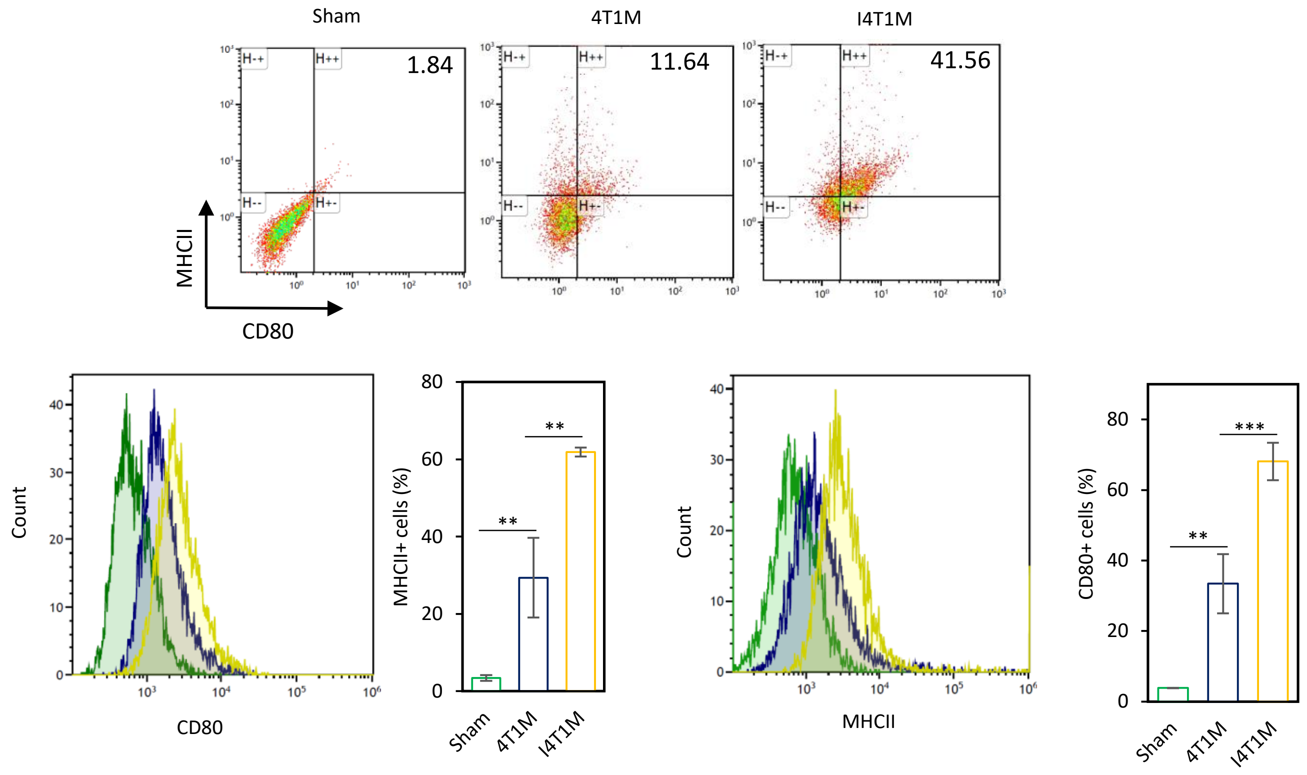

Figure S1. Flow cytometry analysis of maturation markers CD80 and MHCII on RAW264.7 macrophages , with corresponding quantification, after incubation with 4T1M and I4T1M. *$p < 0.05$; **$p < 0.01$; ***$p < 0.001$; ****$p < 0.0001$ by ANOVA.

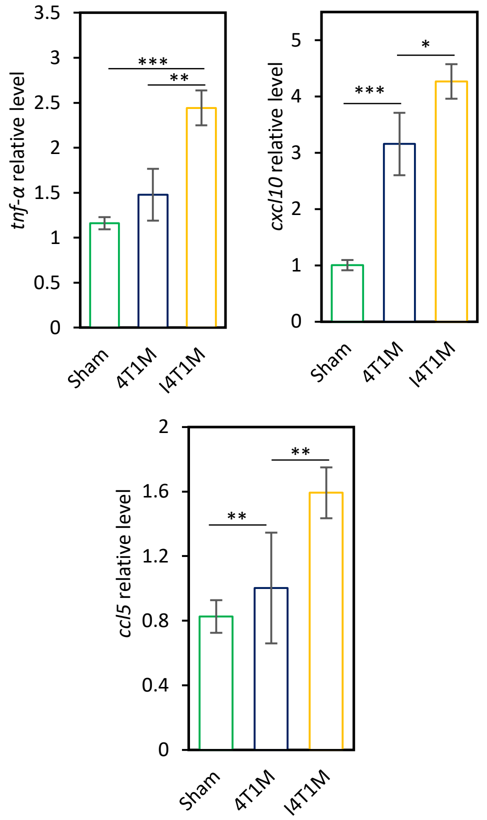

Figure S2. Relative gene expression levels of pro-inflammatory cytokines (*tnf-α*, *cxcl10*, and *ccl5*) in RAW264.7 macrophages following 2 hours of incubation with 4T1M and I4T1M. *$p < 0.05$; **$p < 0.01$; ***$p < 0.001$; ****$p < 0.0001$ by ANOVA.

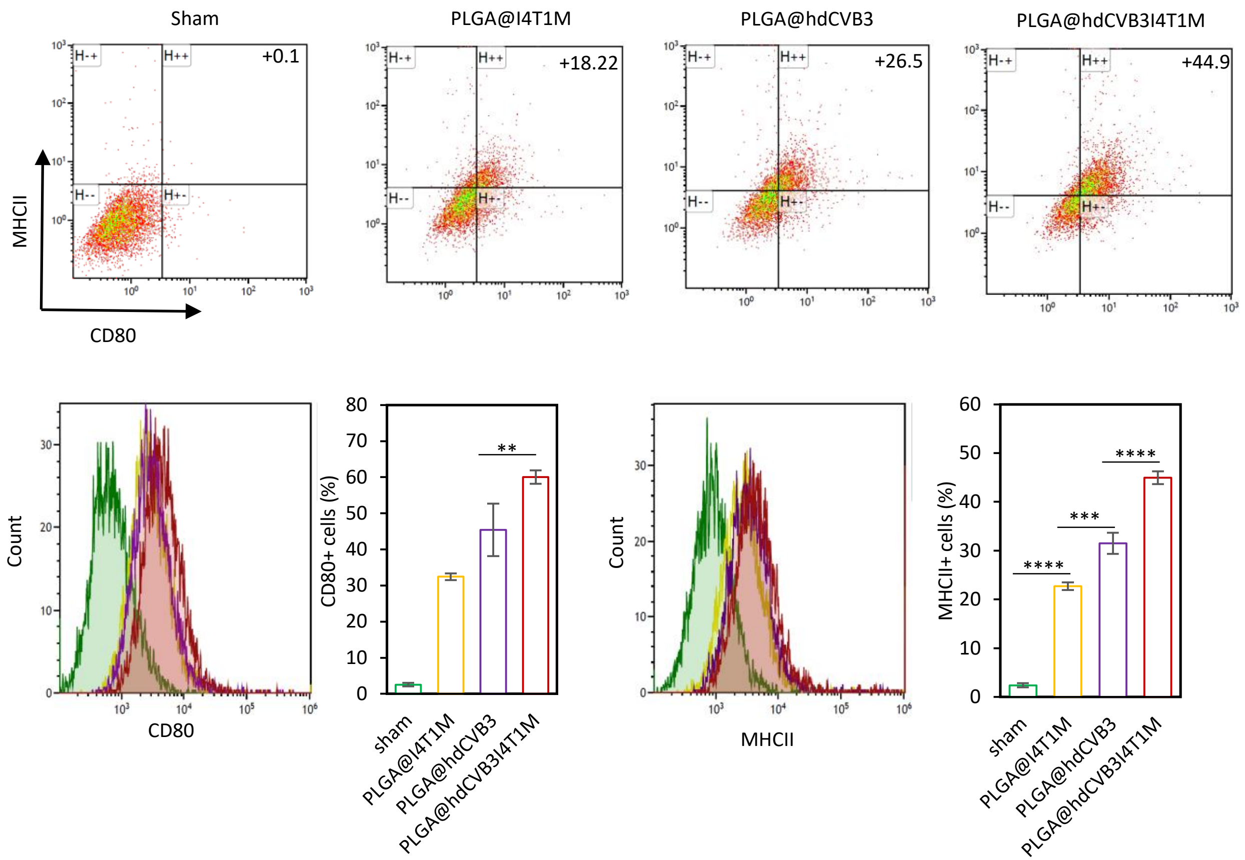

Figure S3. Flow cytometry analysis of the maturation markers CD80 and MHCII on RAW264.7 macrophages after 12-hour incubation with PLGA@I4T1M, PLGA@hdCVB3, and PLGA@hdCVB3I4T1M, with corresponding quantitative data. *$p < 0.05$; **$p < 0.01$; ***$p < 0.001$; ****$p < 0.0001$ by ANOVA.

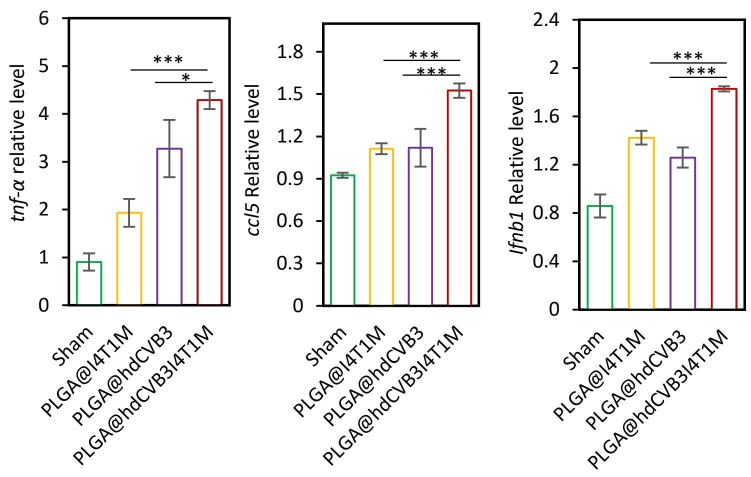

Figure S4. Relative gene expression of pro-inflammatory cytokines (*tnf-α*, *ccl5*, and *ifnb1*) in RAW264.7 macrophages following 2-hour incubation with PLGA@I4T1M, PLGA@hdCVB3, and PLGA@hdCVB3I4T1M. *p < 0.05; **p < 0.01; ***p < 0.001; ****p < 0.0001 by ANOVA.

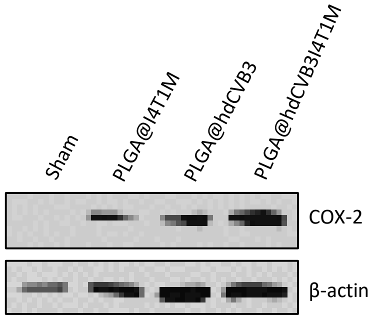

Figure S5. Western blot analysis of COX-2 expression in RAW264.7 macrophages treated with PLGA@I4T1M, PLGA@hdCVB3, and PLGA@hdCVB3I4T1M for 24 hours.

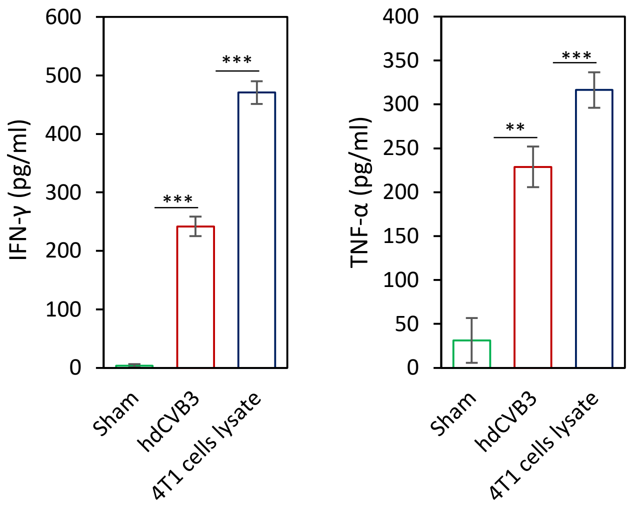

Figure S6. Splenocytes from vaccinated, tumor-free mice were restimulated ex vivo with 4T1 tumor cell lysate, heat-deactivated CVB3 (hdCVB3), or PBS (Sham). ELISA analysis of IFN-γ and TNF-α levels showed significantly increased cytokine secretion in response to both tumor and viral antigens, with 4T1 lysate inducing a stronger response, indicating preferential tumor-specific T cell activation. $*p < 0.05$; $**p < 0.01$; $***p < 0.001$; $****p < 0.0001$ by ANOVA.

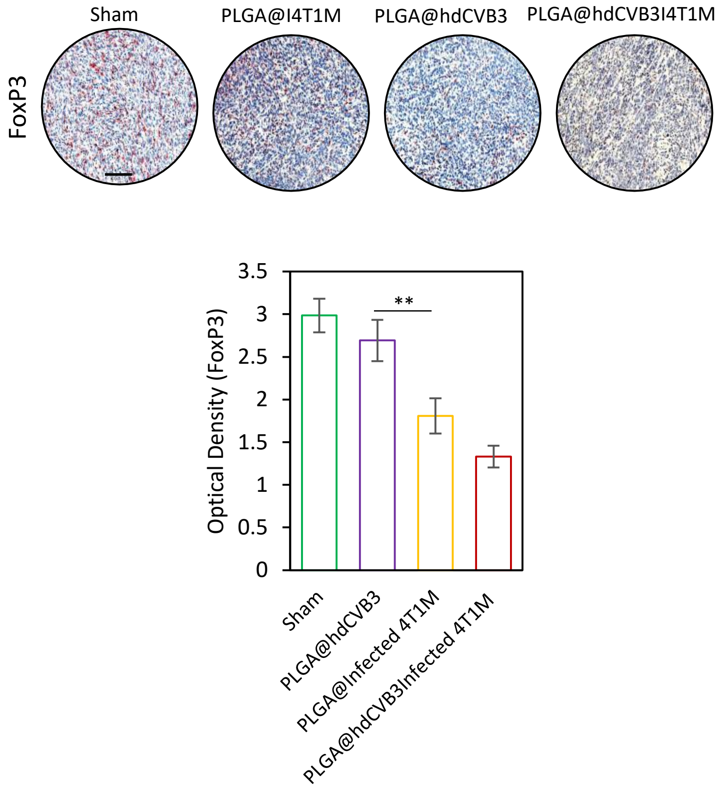

Figure S7. Immunohistochemistry analysis of regulatory T cells in tumor tissues from each treatment group (scale bars: 100 µm), with corresponding quantitative data (n=4). *$p < 0.05$; **$p < 0.01$; ***$p < 0.001$; ****$p < 0.0001$ by ANOVA.

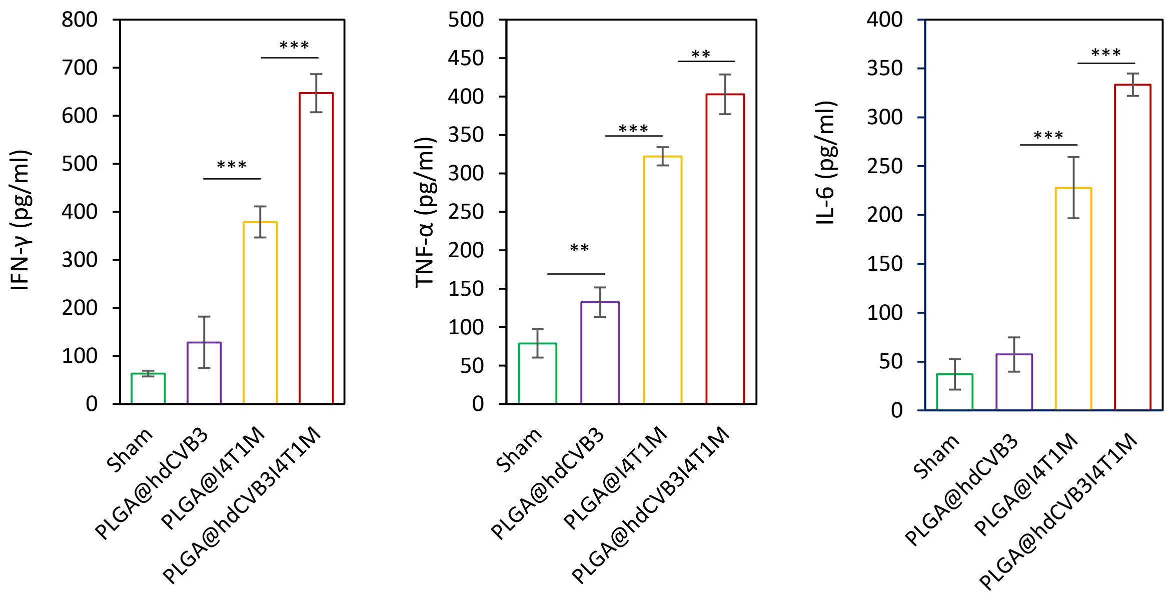

Figure S8. Serum concentrations of TNF-α, IFN-γ, and IL-6 were measured by ELISA following administration of different treatments. All vaccine-treated groups showed elevated cytokine levels compared to controls, with the PLGA@hdCVB3I4T1M group exhibiting the highest levels, indicating robust systemic immune activation (n = 4). *$p < 0.05$; **$p < 0.01$; ***$p < 0.001$; ****$p < 0.0001$ by ANOVA.

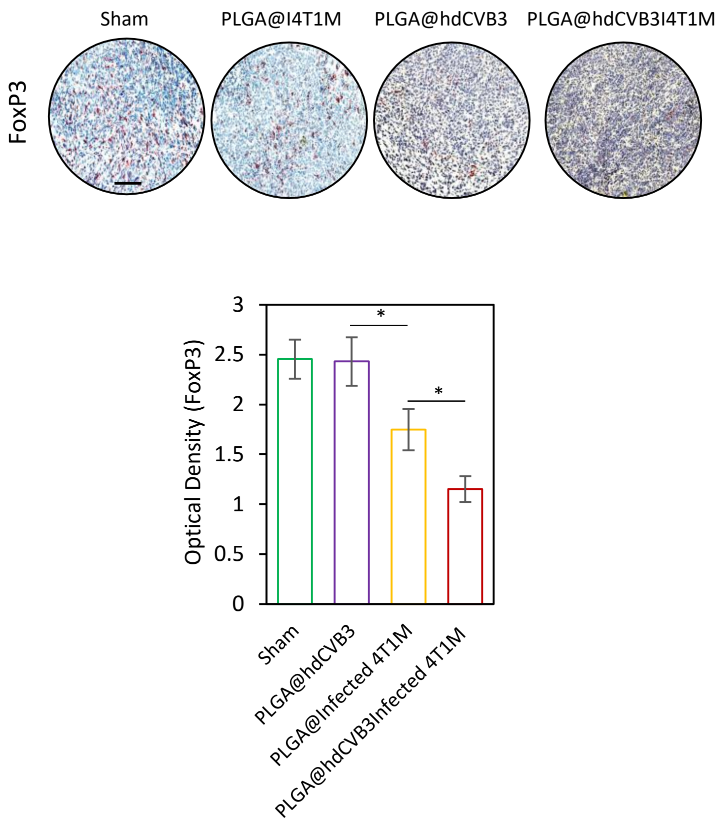

Figure S9. Immunohistochemistry analysis of regulatory T cells in tumor tissues from each treatment group (scale bars: 100 μm), with corresponding quantitative data (n=4). *p < 0.05; **p < 0.01; ***p < 0.001; ****p < 0.0001 by ANOVA.

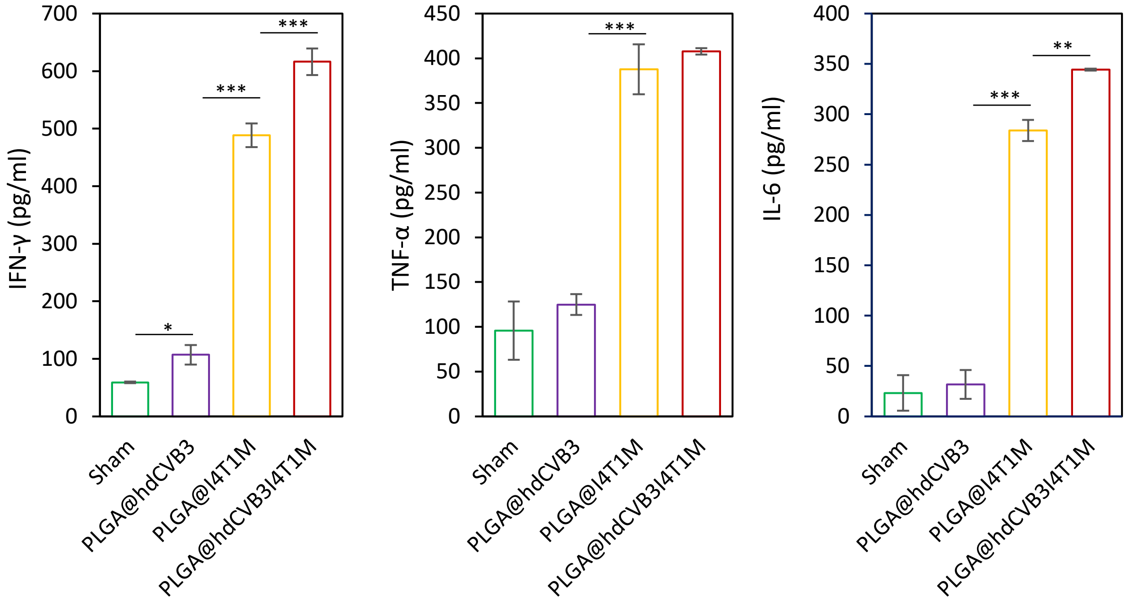

Figure S10. Serum concentrations of TNF-α, IFN-γ, and IL-6 were measured by ELISA following administration of different formulation of vaccines. PLGA@hdCVB3I4T1M group exhibited the highest levels, indicating robust systemic immune activation (n = 4). *$p < 0.05$; **$p < 0.01$; ***$p < 0.001$; ****$p < 0.0001$ by ANOVA.

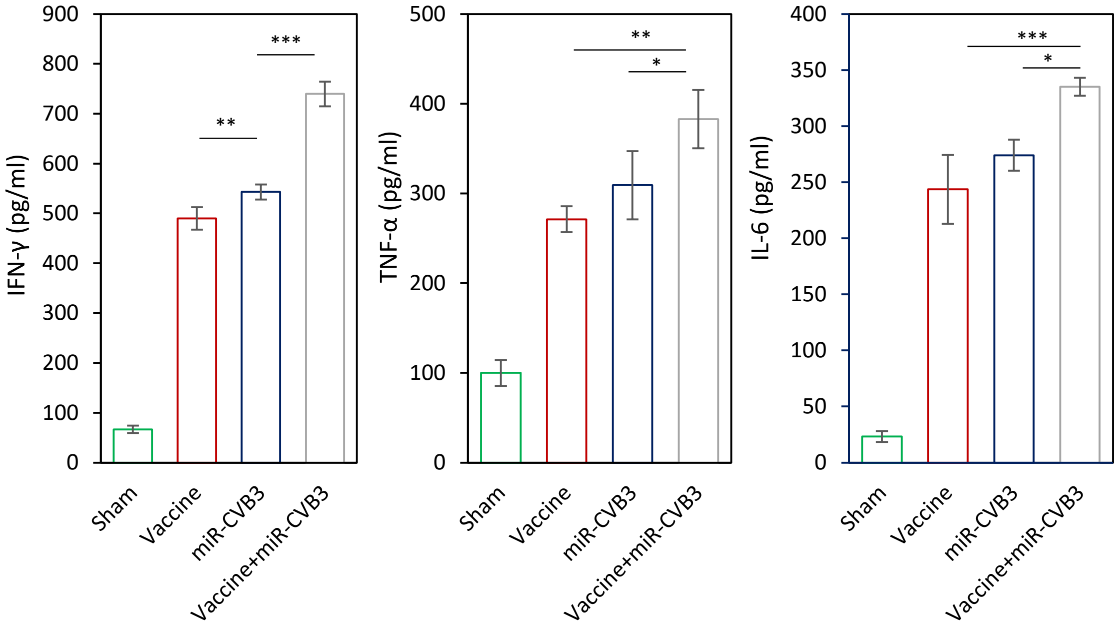

Figure S11. Serum concentrations of TNF-α, IFN-γ, and IL-6 were measured by ELISA following monotherapies and combination therapy. Combination therapy exhibited the highest levels, indicating robust systemic immune activation (n = 4). *$p < 0.05$; **$p < 0.01$; ***$p < 0.001$; ****$p < 0.0001$ by ANOVA.

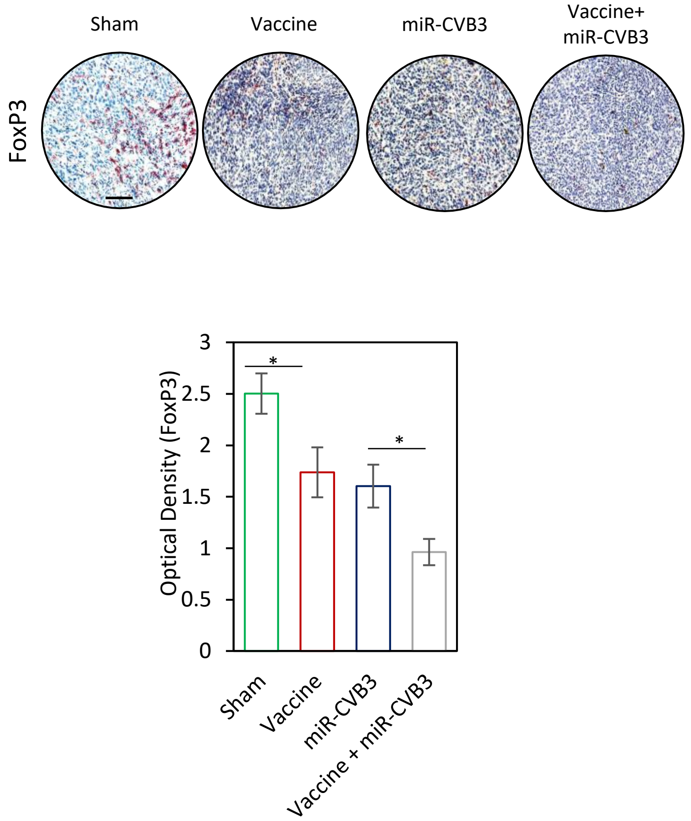

Figure S12. Immunohistochemistry analysis of regulatory T cells in tumor tissues from each treatment group (scale bars: 100 µm), with corresponding quantitative data (n=4). *p < 0.05; **p < 0.01; ***p < 0.001; ****p < 0.0001 by ANOVA.

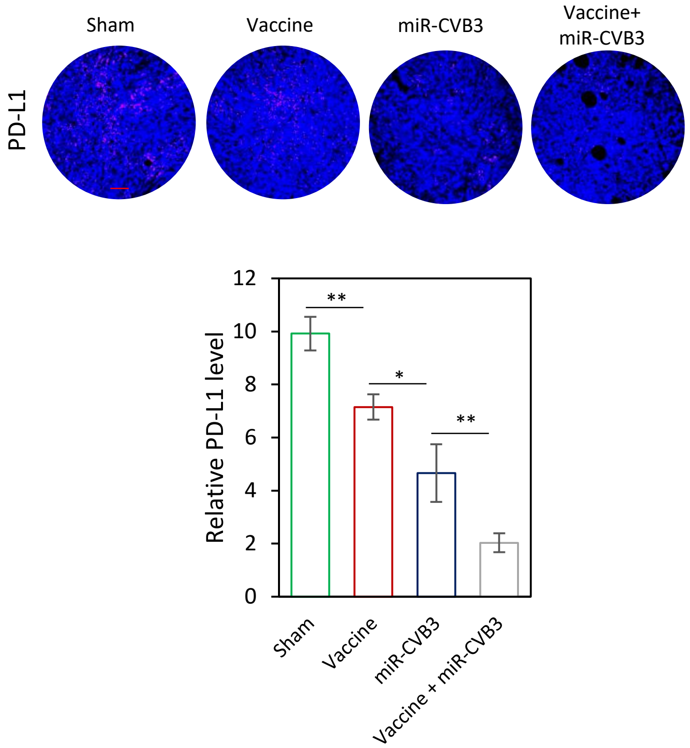

Figure S13. Immunofluorescence analysis of PD-L1 in tumor tissues from each treatment group (scale bars: 50 μm), with corresponding quantitative data (n=4). *p < 0.05; **p < 0.01; ***p < 0.001; ****p < 0.0001 by ANOVA.
